# Elucidating the therapeutic efficacy and mechanisms of arctigenin in ameliorating renal fibrosis: a combined transcriptomic and proteomic study

**DOI:** 10.3389/fphar.2026.1796732

**Published:** 2026-04-23

**Authors:** Yufei Yang, Yiduo Luo, Longshan Zhao, Yukun Bo, Dongdong Zhao, Dan Yang, Jingjing Guo, Xuemiao Yang, Yanan Lv, Yi Tian, Guodong Wu, Ming An

**Affiliations:** 1 Department of Pharmacy, Baotou Medical College, Baotou, Inner Mongolia Autonomous Region, China; 2 Department of Pharmacy, Shenyang Pharmaceutical University, Benxi, Liaoning, China; 3 Department of Academic Affairs, Baotou Medical College, Baotou, Inner Mongolia Autonomous Region, China; 4 Department of Pharmacy, The Fourth Hospital of Baotou, Baotou, Inner Mongolia Autonomous Region, China

**Keywords:** arctigenin, mechanisms, proteomics, renal fibrosis, transcriptomics

## Abstract

**Background:**

Renal fibrosis (RF) is a refractory disease characterized by excessive deposition of extracellular matrix, leading to tissue damage and scar formation. *Fructus arctii* (the dried ripe fruit of *Arctium lappa* L.) is a typical medicinal and edible plant widely utilized in both traditional medicine and culinary practices, rich in lignans, phenolic acids, and dietary fibers. Studies have shown that arctigenin (ATG), the main active component of *F. arctii*, possesses pharmacological effects including anti-inflammatory, anti-fibrotic, and anti-oxidative stress properties. However, the mechanism by which ATG ameliorates RF remains unclear.

**Methods:**

An *in vivo* unilateral ureteral obstruction (UUO) rat model of RF and an *in vitro* TGF-β1-induced HK-2 cell fibrosis model were established. The therapeutic effects of ATG were evaluated through pharmacodynamic experiments. Transcriptomics and proteomics were employed to screen and detect key genes, proteins, and mechanisms involved in ATG-mediated improvement of RF. Finally, core mechanisms were validated both *in vivo* and *in vitro* using experiments such as RT-qPCR, Western blot, immunofluorescence, and flow cytometry.

**Results:**

Both *in vivo* and *in vitro*, ATG improved renal function, alleviated pathological damage, and reduced fibrosis. Combined transcriptomic and proteomic analyses revealed enrichment of pathways related to S100A8/A9, the NF-κB signaling axis, the TCA cycle, and oxidative phosphorylation. Molecular docking demonstrated good binding affinity between ATG and key targets. Further *in vivo* and *in vitro* validation revealed that ATG downregulated the expression of key factors in the S100A8 signaling axis, ameliorated impairments in the TCA cycle and oxidative phosphorylation by inhibiting NF-κB phosphorylation, consequently suppressed the expression of inflammatory and oxidative stress factors, and ultimately downregulated the levels of fibrotic factors by inhibiting the epithelial-mesenchymal transition (EMT) process, thereby improving RF. Using paquinimod, a specific S100A8/A9 inhibitor, we further demonstrated that pharmacological blockade of this pathway recapitulated the anti-fibrotic effects of ATG, providing causal evidence for its functional relevance.

**Conclusion:**

ATG exhibits therapeutic effects in ameliorating RF. Its mechanism involves regulating the TCA cycle and oxidative phosphorylation impairments via the S100A8/A9/NOX/NF-κB signaling pathway, thereby inhibiting inflammation and oxidative stress-driven EMT to improve RF.

## Introduction

Renal fibrosis (RF) is characterized by excessive deposition of extracellular matrix (ECM), leading to tissue scarring and progressive loss of renal function. It represents a common pathological manifestation of various acute and chronic kidney diseases (CKD), affecting an estimated 800 million people worldwide (Huang R. et al., 2023; Bao et al., 2024; Kovesdy, 2022). RF plays a critical role in the continuum from acute kidney injury (AKI) to end-stage renal disease (ESRD), making it a major public health challenge (Arogundade et al., 2020). However, the underlying mechanisms driving RF remain incompletely elucidated, and current treatment options are limited (Abbad et al., 2025). Current clinical management primarily focuses on slowing disease progression using agents such as angiotensin-converting enzyme inhibitors and angiotensin II receptor blockers, including irbesartan (IRB). However, these drugs mostly only slow rather than reverse RF and carry risks such as hyperkalemia and acute renal failure (Banerjee et al., 2022). The number of clinical trials investigating kidney-specific antifibrotic agents remains extremely limited, underscoring the urgent need for novel therapeutic strategies.

Traditional Chinese medicine (TCM), with its multi-target and multi-pathway regulatory capabilities, offers promising avenues for RF treatment (Zhang et al., 2023; Wang et al., 2022). *Fructus arctii*, the dried ripe fruit of *Arctium lappa* L., is a well-known medicinal and edible herb widely utilized in East Asia. Its active component, arctigenin (ATG), a lignan compound, is considered the core active metabolite following oral administration of its glycoside, arctiin (Xie et al., 2003). Previous research has demonstrated that ATG modulates numerous critical biological processes, including inflammatory responses, oxidative stress, fibrogenesis, and apoptosis, showing considerable potential in treating diabetic nephropathy and tissue fibrosis (Wang et al., 2023). Specifically, ATG has been reported to inhibit NF-κB phosphorylation, attenuate endoplasmic reticulum stress, suppress fibroblast activity, and reduce ECM deposition in various disease models, including diabetic nephropathy and fibrotic disorders (Li X. et al., 2024; Zhong et al., 2019; Zhang et al., 2019; Liu et al., 2023; Jiang et al., 2020). However, despite these promising findings, the therapeutic efficacy and underlying mechanisms of ATG in ameliorating RF remain incompletely elucidated, particularly from a multi-omics perspective.

Against this backdrop, the present study aims to investigate the molecular network through which ATG ameliorates RF by integrating transcriptomic and proteomic technologies. Employing multi-omics approaches to explore the multidimensional mechanisms of natural products may provide new insights for both TCM and RF research.

## Materials and methods

### Animals administration

A total of 42 male Sprague-Dawley rats, weighing 200–220 g, were housed in individually ventilated cages within the standard animal facility at the Animal Experiment Center of Baotou Medical College. The housing conditions were maintained at a temperature of 22 °C ± 1 °C, relative humidity of 50% ± 10%, and a 12-h light/dark cycle. All animals were purchased from SPFbio (Animal Production License No. SCXK (JING) 2024–0001). After acclimatization for 1 week with *ad libitum* access to food and water, the rats were randomly divided into seven groups (*n* = 6 per group). The sample size of six rats per group was determined based on a power analysis using preliminary data from our pilot experiments. With an expected effect size of at least 30% reduction in fibrotic markers between the UUO and ATG treatment groups, a standard deviation of 20%, a significance level of *α* = 0.05, and a statistical power of 80%, a minimum of five rats per group was required. To account for potential dropouts or technical failures, six rats per group were enrolled. This sample size is also consistent with previous studies investigating therapeutic interventions in the UUO model of RF, ensuring comparability with established literature while adhering to the principles of reduction in animal experimentation. RF was induced via unilateral ureteral obstruction (UUO) as previously described (Liu et al., 2024). Briefly, under anesthesia induced by intraperitoneal injection of 40 mg/kg sodium pentobarbital, the left ureter was exposed following disinfection and layer-by-layer dissection. The ureter was ligated at the upper and lower one-third points using 4–0 suture, and transected between the ligations to prevent urinary reflux. Muscle and skin layers were subsequently sutured, and animals were kept warm during recovery. Sham-operated animals underwent identical surgical procedures except for ureteral ligation and transection. ATG (HPLC purity ≥98%) was procured from Alfabio (AB0246; Sichuan, China), and IRB was obtained from Huahaipharm (H20030016; Zhejiang, China). The rats were administered the following treatments: low-dose ATG (1 mg/kg/day; ATG-L group), medium-dose ATG (3 mg/kg/day; ATG-M group), and high-dose ATG (9 mg/kg/day; ATG group). The dosages of ATG (1, 3, and 9 mg/kg/day) were selected based on previous pharmacological studies demonstrating its anti-fibrotic and anti-inflammatory effects in a rat model of renal injury (Li et al., 2017). These doses were also chosen to cover a range that would allow assessment of dose-dependent responses, with the high dose approximating the human equivalent dose based on body surface area conversion. Preliminary experiments in our laboratory confirmed that these doses were well-tolerated and did not cause overt toxicity, as evidenced by normal behavior, body weight gain, and survival rates throughout the experimental period. The positive control group (IRB group) received irbesartan at 10 mg/kg/day, a dose equivalent to the human equivalent dose based on body surface area conversion. A combination therapy group (ATG + IRB group) received both ATG (9 mg/kg/day) and IRB (10 mg/kg/day). Both Sham and UUO groups received equal volumes of physiological saline. Drug interventions commenced 1 day after surgery and continued for 3 weeks. All rats were euthanized by intraperitoneal injection of pentobarbital sodium at a dose of 150 mg/kg. Thereafter, blood samples and the obstructed kidneys were collected for subsequent analyses.

### Body weight, survival, and renal index analysis

Body weight was measured daily, and survival status was recorded to analyze trends in weight change and survival rates. Following extraction and cleaning, the obstructed kidney was photographed for documentation, then weighed and recorded. The renal index (%) was calculated as follows: (weight of the obstructed kidney in grams/rat body weight in kilograms) × 100%.

### Histopathologic evaluation of kidney tissues

All reagents used for histopathological analysis were procured from Servicebio (Hubei, China). Renal tissue samples were fixed in 4% paraformaldehyde solution (G1101) for 24 h and subsequently embedded in paraffin to prepare tissue sections. Following deparaffinization and rehydration, sections were stained with Hematoxylin and Eosin (HE, G1005) and Masson’s trichrome (G1006). After dehydration and mounting, pathological alterations in renal tissues were randomly examined using an Eclipse microscope (Nikon; Tokyo, Japan). HE-stained sections were evaluated according to the Remuzzi scoring system (Remuzzi et al., 2006). Specifically, within a single microscopic field, the following criteria were applied: One point was assigned for glomerular fibrosis, tubular atrophy, or interstitial fibrosis affecting less than 20% of the field, or for vascular wall thickening occupying less than half of the lumen; Two points were assigned for involvement of 20%–50% of the field, or wall thickening equal to or slightly exceeding half the lumen; Three points were assigned for involvement exceeding 50% of the field, or wall thickening greater than half the lumen; No points were recorded in the absence of pathological changes. The total score was calculated as the sum of individual category scores. For Masson’s trichrome-stained sections, a randomly selected field was analyzed using ImageJ software (v1.54; https://imagej.net/ij/index.html). The collagen volume fraction (CVF) was determined as: (fibrotic area/total tissue area) × 100%

### Transcriptome sequencing and data analysis

Total RNA samples that passed quality control were first treated with DNase I to remove genomic DNA contamination. The mRNA was then enriched using Oligo(dT) magnetic beads. The enriched mRNA was fragmented, and first-strand cDNA was synthesized using random primers. During second-strand synthesis, dUTP was incorporated instead of dTTP for strand marking. The double-stranded cDNA underwent end repair, poly(A) tailing, and adapter ligation. The second strand, containing dUTP markers, was digested using UDG enzyme. The products were then amplified by PCR and purified. After library quality validation, the PCR products were denatured into single strands and circularized. Any remaining linear DNA molecules were digested. The circularized products were subsequently subjected to rolling circle amplification to generate DNA nanoballs (DNBs). All procedures and related reagents were supplied by Beijing Genomics Institution (Guangdong, China). Sequencing was performed on the DNBSEQ platform (MGI; Guangdong, China) with PE150 read length. Raw data were processed using SOAPnuke (v1.5.6; https://github.com/BGI-flexlab/SOAPnuke) to obtain clean data. The clean reads were aligned to the reference genome using HISAT2 (v2.2.1; https://daehwankimlab.github.io/hisat2). Bowtie2 (v2.3.4.3; http://bowtie-bio.sourceforge.net/bowtie2/index.shtml) was employed to align the clean data to the reference gene set. Gene expression quantification was performed with RSEM (v1.3.1; https://deweylab.github.io/RSEM). Differential gene expression analysis was conducted using DESeq2 (v1.4.5; https://bioconductor.org/packages/3.16/bioc/html/DESeq2.html). Significantly differentially expressed genes were subsequently subjected to Gene Ontology (GO; http://www.geneontology.org) and Kyoto Encyclopedia of Genes and Genomes (KEGG; https://www.kegg.jp) enrichment analyses.

#### Proteomic profiling

Renal tissues were homogenized via ultrasonication in lysis buffer, and protein concentration was determined using a BCA assay kit (P0012, Beyotime; Shanghai, China). Equal amounts of protein from each sample were digested with trypsin (V5111, Promega; WI, USA) to generate peptides. Data acquisition was performed using a timsTOF Pro mass spectrometer (Bruker) operating in dia-PASEF mode. The ion source voltage was set to 1.75 kV, and the primary mass spectrometry scanning range was set from 300 to 1500 m*/z*. Secondary mass spectrometry scanning was conducted within the range of 400–850  m*/z*, using a window of 7  m*/z*. Raw data were processed with the DIA-NN search engine (v1.8; https://github.com/vdemichev/DiaNN) for protein identification and matching. Following quality control, normalized intensity values were centralized to obtain relative protein quantification across samples. Differentially expressed proteins were subsequently screened, and GO and KEGG enrichment analyses were performed.

### Expression pattern clustering, gene set enrichment analysis (GSEA), and integrated transcriptomics-proteomics analysis

Differentially expressed genes and proteins were subjected to Fuzzy C-Means Clustering for expression pattern analysis using the R package TCseq (v1.8.0; https://bioconductor.org/packages/TCseq). Gene Set Enrichment Analysis (GSEA) was performed using the R package clusterProfiler (v3.10.1; https://bioconductor.org/packages/clusterProfiler). To systematically integrate the transcriptomic and proteomic datasets, we first mapped quantifiable protein accessions to their corresponding transcript IDs using the UniProtKB–Ensembl cross-reference database, thereby retaining only those features that were quantifiable in both omics layers for subsequent analysis. Differential expression analysis was performed separately for each dataset using consistent statistical criteria to ensure comparability across omics layers. For both transcriptomic and proteomic data, features with |log_2_(fold change)| >1 and Benjamini–Hochberg adjusted *p* < 0.05 were considered significantly differentially expressed. The integrated analysis then proceeded with correlation analysis between protein and transcript expression changes to assess global concordance across the two omics layers. Overlapping significantly changed genes and proteins were identified by comparing the differential expression results from both datasets. Finally, these overlapping targets were subjected to GO and KEGG pathway enrichment analyses with a significance threshold of *p* < 0.05, aiming to pinpoint key molecular events at both transcriptional and translational levels that may underlie the therapeutic effects of ATG in ameliorating RF.

#### Molecular docking

The structure of ATG was obtained in SDF format from the PubChem database (https://pubchem.ncbi.nlm.nih.gov) and subsequently converted into MOL2 format using Open Babel (v2.4.1; https://openbabel.org) to prepare the ligand for molecular docking. The protein structure used as the receptor was retrieved from the PDB database (https://www.rcsb.org) in PDB format. Molecular docking was performed using AutoDockTools (v1.5.7; https://autodocksuite.scripps.edu/adt). Preprocessing steps included preparation of both ligand and receptor structures by removing water molecules and adding hydrogen atoms, followed by definition of the binding pocket coordinates. Docking simulations were carried out using the Genetic Algorithm method, and the conformation with the most favorable binding energy was recorded. The resulting docking poses were visualized using PyMOL (v2.5.7; https://pymol.org) and BIOVIA Discovery Studio Visualizer (v4.5; https://www.3ds.com/products/biovia/discovery-studio/visualization).

### Cell culture and intervention

Unless otherwise specified, all cell culture reagents were purchased from Servicebio. Human renal cortical proximal tubular epithelial HK-2 cells (STCC10303P, Servicebio) were maintained in a humidified incubator (Thermo Fisher; MA, USA) at 37 °C with 5% CO_2_, and cultured in DMEM/F-12 medium (G4612) supplemented with 10% fetal bovine serum (G8003) and 1% penicillin-streptomycin (G4003). Cells were subsequently divided into six groups. With the exception of the Control group, which received only complete DMEM/F-12 medium, all other groups were treated with 10 ng/mL TGF-β1 (BH00005, Proteintech; IL, USA) to establish a fibrosis model. After 24 h of TGF-β1 stimulation, the TGF-β1 group was switched to complete DMEM/F-12 medium for continued culture. The remaining groups then began drug treatment, with all dosages selected based on cell viability assays and preliminary experiments. The L-ATG group received low-dose ATG at 1 mg/mL (2.69 mM), the H-ATG group received high-dose ATG at 2 mg/mL (5.37 mM), the PAQ group was treated with paquinimod (PAQ) (GC31346, GlpBio; CA, USA) at 125 μg/mL (356.72 μM), and the H-ATG + PAQ group received a combination of ATG (2 mg/mL) and PAQ (125 μg/mL). All cells were harvested 24 h post-treatment for subsequent experiments.

#### Cell viability analysis

HK-2 cells were seeded into 96-well plates at a density of 1 × 10^4^ cells per well and cultured for 24 h under standard conditions (37 °C, 5% CO_2_) to allow for cell attachment and stabilization. Following this incubation period, the cells were treated with various concentrations of the test compounds for an additional 24 h. After treatment, the culture medium was carefully removed, and each well was supplemented with 10 μL of CCK-8 solution (MA0218, MeilunBio; Liaoning, China). The plates were then incubated at 37 °C with 5% CO_2_ for 1 h in the dark. The optical density (OD) of each well was measured at a wavelength of 450 nm using a Multiskan GO microplate reader (Thermo Fisher). Cell viability was calculated according to the following formula: ((experimental well - blank well)/(control well - blank well))×100%。

### Wound healing assay

HK-2 cells were seeded into 6-well plates at a density of 5 × 10^5^ cells per well and cultured for 24 h under standard conditions (37 °C, 5% CO_2_) to facilitate cell adherence. After the cells formed a confluent monolayer, a straight scratch was introduced in each well using a 10 μL pipette tip. The wells were gently rinsed three times with PBS solution (G4202, Servicebio) to remove detached cells, followed by the application of designated drug treatments according to the experimental grouping. Images of the scratch were immediately captured at 0 h using an Eclipse microscope, after which the plates were returned to the incubator for continued culture. After 24 h, the same visual fields were re-located and photographed again. The scratch areas were quantified using ImageJ software, and the wound healing rate was calculated as: ((0 h scratch area - 24 h scratch area)/0 h scratch area)×100%。

#### Flow cytometry

The intracellular reactive oxygen species (ROS) levels were measured using a commercial ROS assay kit (MA0219, MeilunBio). Following cell seeding and drug treatment in 6-well plates under conditions analogous to the scratch assay, cells were detached using trypsin (G4004, Servicebio) and centrifuged at 500 *g* for 5 min using an LSC centrifuge (Sunne; Shanghai, China). The resulting cell pellet was resuspended in PBS. Cells were then loaded with 10 μM DCFH-DA probe at a density of 1 × 10^7^ cells per mL and incubated in the dark for 30 min at 37 °C under 5% CO_2_. After incubation, cells were washed with culture medium and resuspended. Fluorescence intensity was analyzed using a CytoFLEX flow cytometer (Beckman Coulter; CA, USA) with the FITC channel selected for detection.

#### Immunofluorescence

HK-2 cells were seeded into 6-well plates containing pre-placed cell climbing sheets, following the identical procedures for cell inoculation and drug treatment as described in the preceding section. Unless otherwise specified, all reagents for immunofluorescence assays were obtained from Servicebio. Cell climbing sheets were fixed with 4% paraformaldehyde fixative (G1101) for 15 min, followed by antigen retrieval in antigen retrieval buffer (G1202) using microwave boiling for 10 min. After three washes with PBS and slight drying, the sheets were blocked by applying 3% BSA (GC305006) at room temperature for 30 min. Primary antibodies against NOX2 (GB112362, 1:1000), IKKβ (GB115540, 1:150), and IκBα (10268-1-AP, 1:500; Proteintech) were applied and incubated overnight at 4 °C. After circling the sample area with a hydrophobic pen, FITC-conjugated secondary antibody (GB22303, 1:100) was applied to cover the cell sheet and incubated at room temperature for 50 min. Following incubation, excess liquid was gently removed, and DAPI staining solution (G1012) was applied for 10 min at room temperature. After three additional PBS washes, autofluorescence quencher (G1221) was applied for 5 min. The sheets were washed a final time with PBS and mounted. Images were captured using an Eclipse microscope and analyzed for fluorescence intensity with ImageJ software.

### Renal function, renal fibrosis, oxidative stress, TCA cycle, and oxidative phosphorylation assays

Reagent kits for assessing renal function, including blood urea nitrogen (BUN, G1201W), serum creatinine (SCr, G1204W), and serum uric acid (SUA, G1202W), were employed. Oxidative stress markers were evaluated using kits for malondialdehyde (MDA, G0109W), superoxide dismutase (SOD, G0101W), and reduced glutathione (GSH, G0206W). The tricarboxylic acid (TCA) cycle was determined with citric acid (CA, G0864W), NAD-malic enzyme (NAD-ME, G0819W), and NAD-malate dehydrogenase (NAD-MDH, G0821W) kits. Oxidative phosphorylation capacity was assessed via kits for adenosine triphosphate (ATP, G0857W), creatine kinase (CK, G0855W), and NAD kinase (NADK, G0854W). All aforementioned kits were procured from Gracebio (Jiangsu, China). Additionally, reagent kits for evaluating RF markers, including α-smooth muscle actin (α-SMA, ml038078), fibronectin (ml302818), and collagen I (ml058800), were obtained from Mlbio (Shanghai, China). All experimental procedures involving these kits were strictly performed in accordance with the manufacturers’ instructions.

#### Quantitative real-time reverse-transcription PCR (RT-qPCR)

Unless otherwise specified, all reagents and custom primer syntheses for RT-qPCR experiments were procured from Servicebio. Total RNA was extracted using RNA Extraction Solution (G3013), and reverse transcription into cDNA was carried out with SweScript All-in-One RT SuperMix for qPCR (G3337). Quantitative PCR was performed using a StepOnePlus Real-Time PCR System (Thermo Fisher), SYBR Green qPCR Master Mix (G3326), and gene-specific primers (Table 1). The thermocycling conditions were set as follows: initial denaturation at 95 °C for 30 s, followed by 40 cycles of denaturation at 95 °C for 15 s, and annealing/extension at 60 °C for 30 s. The relative mRNA expression levels of target genes were calculated using the 2^−ΔΔCT^ method (Livak and Schmittgen, 2001).

**TABLE 1:** Information of primers used for RT-qPCR in the study.

Gene	Accession number	Species	Forward primer (5′-3′)	Reverse primer (5′-3′)
α-SMA	NM_031004.2	Rat	ACC​ATC​GGG​AAT​GAA​CGC​TT	CTG​TCA​GCA​ATG​CCT​GGG​TA
	NM_001141945.2	Human	CAA​TGT​CCT​ATC​AGG​GGG​CAC	CGG​CTT​CAT​CGT​ATT​CCT​GTT
Collagen I	NM_053304.1	Rat	GGC​AAG​AAC​GGA​GAT​GAT​GG	ACC​ATC​CAA​ACC​ACT​GAA​ACC​TC
	NM_000088.3	Human	TGG​CAA​AGA​TGG​ACT​CAA​CG	TCA​CGG​TCA​CGA​ACC​ACA​TT
Fibronectin	NM_019143.2	Rat	AAA​CCT​CTA​CGG​GTC​GCT​G	GCG​CTG​GTG​GTG​AAG​TCA​AA
	NM_001306129.1	Human	GCA​TTG​CCA​ACC​TTT​ACA​GAC​C	TTG​GAA​ATG​TGA​GAT​GGC​TGT​G
Vimentin	NM_031140.1	Rat	GCA​AAG​CAG​GAG​TCA​AAC​GA	CTT​CCT​TCA​TGT​TCT​GGA​TCT​CAT​C
	NM_003380.5	Human	TGC​CGT​TGA​AGC​TGC​TAA​CTA	ATC​CTG​CTC​TCC​TCG​CCT​T
E-cadherin	NM_031334.1	Rat	ATG​AGG​TCG​GTG​CCC​GTA​TT	CGT​TGG​TCT​TGG​GGT​CTG​TGA
	NM_004360	Human	GAG​AAC​GCA​TTG​CCA​CAT​ACA​C	GAG​CAC​CTT​CCA​TGA​CAG​ACC​C
N-cadherin	NM_031333.2	Rat	TAT​GAT​GAA​GAA​GGT​GGA​GGA​GAG	TAC​TGT​GGC​TCA​GCG​TGG​ATA​G
	NM_001308176.2	Human	AAG​AGG​CAG​AGA​CTT​GCG​AAA​C	TGG​AGT​CAC​ACT​GGC​AAA​CCT​T
TNF-α	NM_012675.3	Rat	CCA​CCA​CGC​TCT​TCT​GTC​TAC​TG	TGG​GCT​ACG​GGC​TTG​TCA​CT
	NM_000594.3	Human	GCT​GCA​CTT​TGG​AGT​GAT​CG	ATG​AGG​TAC​AGG​CCC​TCT​GA
IL-6	NM_012589.2	Rat	AAG​AGA​CTT​CCA​GCC​AGT​TGC​C	TGT​GGG​TGG​TAT​CCT​CTG​TGA​AG
	NM_000600.5	Human	ATG​AGG​AGA​CTT​GCC​TGG​TGA​A	CTC​TGG​CTT​GTT​CCT​CAC​TAC​TCT​C
IL-1β	NM_031512.2	Rat	TGT​GAC​TCG​TGG​GAT​GAT​GAC	CCA​CTT​GTT​GGC​TTA​TGT​TCT​GTC
	NM_000576.2	Human	CGA​TCA​CTG​AAC​TGC​ACG​CTC	ACA​AAG​GAC​ATG​GAG​AAC​ACC​ACT​T
S100A8	NM_053822.2	Rat	GTG​CCC​TCA​GTT​TGT​GCA​GAA​TA	TTA​CTC​CTT​GTG​GCT​GTC​TTT​ATG
	NM_001319196.1	Human	ATG​TTG​ACC​GAG​CTG​GAG​AAA	CAC​GCC​CAT​CTT​TAT​CAC​CAG
S100A9	NM_053587.2	Rat	GAC​ATC​CTG​ACA​CCC​TGA​ACA​AG	CCC​ATC​AGC​ATC​ATA​CAC​TCC​TC
	NM_002965.4	Human	AGC​TGG​AAC​GCA​ACA​TAG​AGA​C	TCA​GCT​GCT​TGT​CTG​CAT​TTG​T
NOX2	NM_023965.1	Rat	AGG​TGG​TGA​TGT​TAG​TGG​GAG​C	TGT​TTC​TTT​CTT​GCA​TCT​GGG​T
	NM_000397.4	Human	AGA​GCC​AGA​TGC​AGG​AAA​GGA	GGA​GAT​GCT​TTG​TTT​ACT​CAG​GG
NOX4	NM_053524.1	Rat	CGC​ACA​GTC​CTG​GCT​TAC​CT	ACC​ACC​ACC​ATG​CAG​ACA​CC
	NM_001143836.3	Human	CTT​GGC​TTT​GGA​TTT​CTG​GAC​C	GAC​ACA​TTG​TGA​GGG​TAA​ATG​GAT​G
IκBα	NM_001105720.2	Rat	CCC​AAG​TAC​CCG​GAT​ACA​GCA	GTC​ATC​GTA​GGG​CAA​CTC​ATC​TT
	NM_020529.3	Human	AAA​GAC​GAG​GAG​TAC​GAG​CAG​AT	CAG​GTT​GTT​CTG​GAA​GTT​GAG​GA
NF-κB p65	NM_199267.2	Rat	ATT​AGC​CAG​CGC​ATC​CAG​AC	ATC​TTG​AGC​TCG​GCA​GTG​TT
	NM_001145138.2	Human	ACC​GGA​TTG​AGG​AGA​AAC​GTA	TCT​GCC​CAG​AAG​GAA​ACA​CC
GAPDH	NM_017008.4	Rat	CTG​GAG​AAA​CCT​GCC​AAG​TAT​G	GGT​GGA​AGA​ATG​GGA​GTT​GCT
β-actin	NM_001101.5	Human	CAC​CCA​GCA​CAA​TGA​AGA​TCA​AGA​T	CCA​GTT​TTT​AAA​TCC​TGA​GTC​AAG​C

#### Western blot analysis

Protein extraction was performed using RIPA lysis buffer (G2002, Servicebio) supplemented with PMSF (SL1079, Coolaber; Beijing, China) and a phosphatase inhibitor cocktail (G2007, Servicebio). Protein concentration was determined using a BCA assay kit (ZJ102, Epizymebio; Shanghai, China). An equal amount of 20 μg protein per lane was separated by SDS-PAGE and transferred onto PVDF membranes (FFP28, Beyotime). The membranes were then blocked with 5% skim milk (LP0033B, Solarbio; Beijing, China) for 2 h at room temperature. Following blocking, the membranes were incubated overnight at 4 °C with the following primary antibodies: α-SMA (PTM-5216, 1:1000, PTMbio; Zhejiang, China), Vimentin (PTM-5376, 1:1000, PTMbio), Collagen I (PTM-6219, 1:1000, PTMbio), S100A8 (15792-1-AP, 1:1000, Proteintech), S100A9 (26992-1-AP, 1:2000, Proteintech), NF-κB p65 (10745-1-AP, 1:3000, Proteintech), p-NF-κB p65 (82335-1-RR, 1:6000, Proteintech), and β-actin (66009-1-Ig, 1:60,000, Proteintech). After washing three times with TBST buffer (G0004, Servicebio), the membranes were incubated for 1.5 h at room temperature with HRP-conjugated goat anti-mouse (SA00001-1, 1:6000, Proteintech) or anti-rabbit (SA00001-2, 1:6000, Proteintech) secondary antibodies. Finally, protein bands were visualized using an ECL kit (G2020, Servicebio) on a 5200T chemiluminescence imaging system (Tanon; Shanghai, China), and relative protein expression levels were analyzed using ImageJ software.

### Statistical analysis

Statistical analyses in this study were performed using GraphPad Prism software (v9.5.0; https://www.graphpad.com). All quantitative data are expressed as mean ± standard error of the mean (SEM). Comparisons between two groups were assessed using Student’s t-test, while one-way analysis of variance (ANOVA) was applied for comparisons across multiple groups. A *p* value of less than 0.05 was considered statistically significant.

## Results

### ATG ameliorates renal function and fibrosis in a dose-dependent manner

**FIGURE 1:**
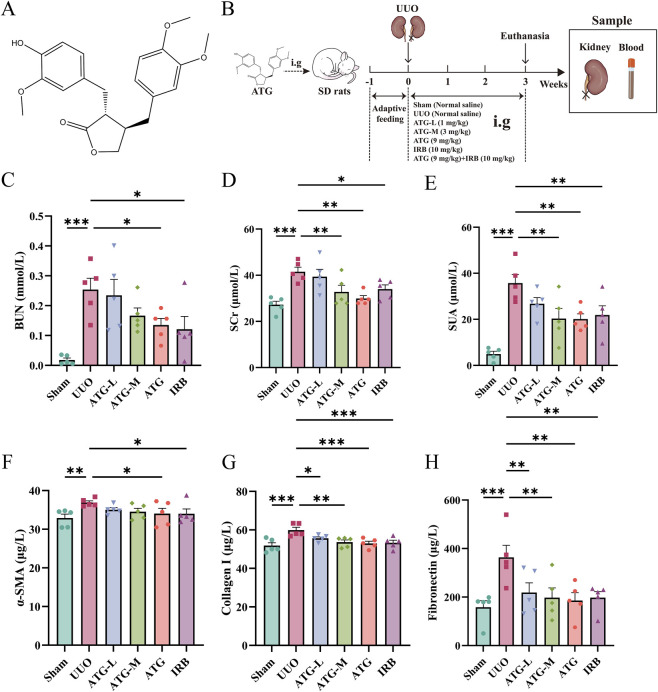
ATG ameliorates renal function and fibrosis in a dose-dependent manner. **(A)** Chemical structure of ATG. **(B)** Schematic of the animal experimental protocol. **(C)** Serum blood urea nitrogen (BUN) levels. **(D)** Serum creatinine (SCr) levels. **(E)** Serum uric acid (SUA) levels. **(F)** Expression of α-smooth muscle actin (α-SMA) in renal tissue. **(G)** Expression of collagen I in renal tissue. **(H)** Expression of fibronectin in renal tissue. Data are presented as mean ± SEM, *n* = 5 per group, *p < 0.05, **p < 0.01, ***p < 0.001.

Rats were administered varying concentrations of ATG (Figure 1A) along with IRB as a positive control. Renal tissues and blood samples from the obstructed side were collected according to the experimental timeline outlined in Figure 1B. Compared with the Sham group, the UUO group exhibited significantly elevated levels of BUN, SCr, and SUA, indicating impaired renal function (*p <* 0.001) (Figures 1C–E). In contrast, the IRB (BUN, *p =* 0.013; SCr, *p =* 0.039; SUA, *p =* 0.006), ATG-L, and ATG-M (SCr, *p =* 0.01; SUA, *p =* 0.003) groups—particularly the high-dose ATG group (BUN, *p =* 0.024; SCr, *p =* 0.001; SUA, *p =* 0.002)—showed markedly reduced levels of these renal function markers relative to the UUO group. Furthermore, elevated expression of α-SMA (*p =* 0.008), collagen I (*p <* 0.001), and fibronectin (*p <* 0.001) in the UUO group confirmed successful induction of RF, whereas all drug-treated groups reversed these aberrant increases (*p <* 0.05), suggesting the therapeutic potential of ATG in ameliorating RF (Figures 1F–H). Notably, the extent of suppression in both renal dysfunction and fibrotic markers correlated with increasing ATG dosage, indicating a clear dose-dependent response. The high-dose ATG group (9 mg/kg) demonstrated significant improvements in both renal function and fibrosis parameters (α-SMA, *p =* 0.047; Collagen I, *p <* 0.001; Fibronectin, *p =* 0.002). Consequently, this dosage was selected for subsequent experimental investigations.

### ATG ameliorates RF induced by UUO in rats

**FIGURE 2:**
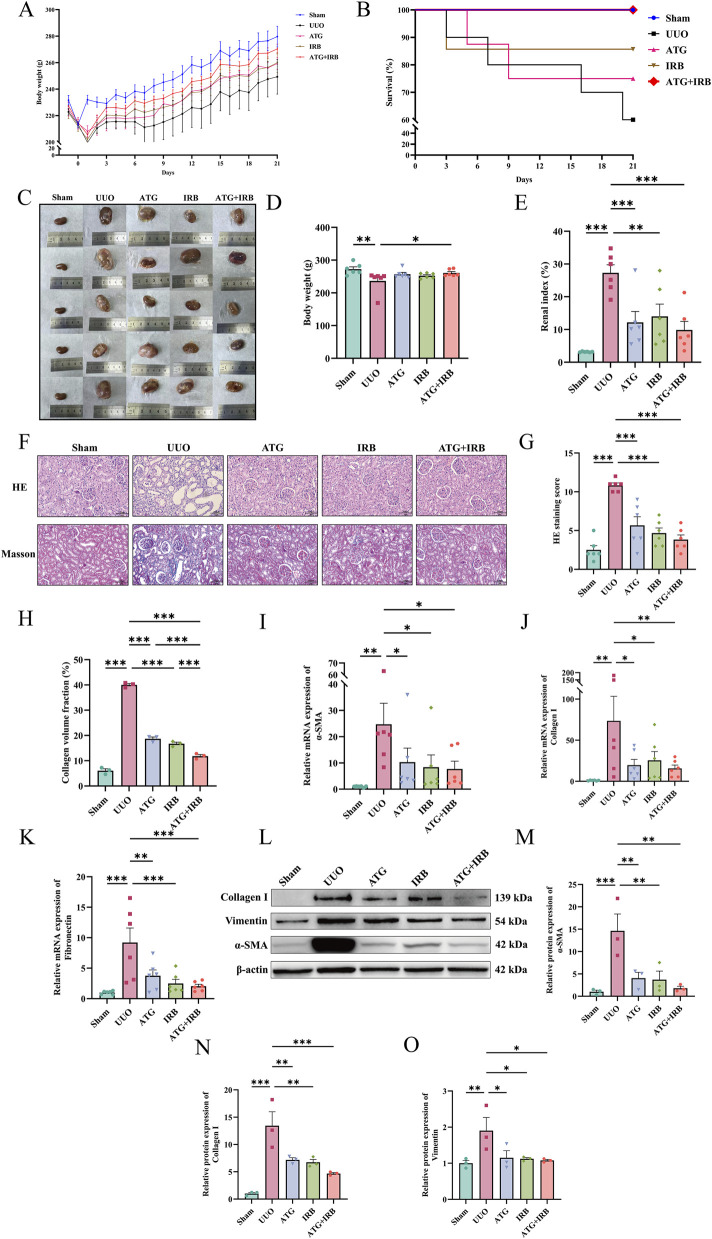
ATG exhibits therapeutic potential in ameliorating UUO-induced RF in rats. **(A)** Changes in body weight of rats during the experimental period. **(B)** Survival rates of rats throughout the study. **(C)** Morphological observations of the obstructed kidneys. **(D)** Body weight of rats on the day of tissue collection. **(E)** Renal index calculated for each group. **(F–H)** Representative images of HE and Masson staining of renal tissues (scale bar = 100 μm), accompanied by quantitative analysis of HE scores and collagen volume fraction. **(I–K)** Relative mRNA expression levels of α-SMA, collagen I, and fibronectin in renal tissues as determined by RT-qPCR. **(L–O)** Western blot results and quantitative analysis of relative protein expression levels of α-SMA, collagen I, and vimentin in renal tissues. Data are presented as mean ± SEM, *n* = 6 per group (*n* = 3 per group for Western blot and Masson staining), *p < 0.05, **p < 0.01, ***p < 0.001.

To further investigate the therapeutic potential of ATG in alleviating RF, we initially assessed several fundamental physiological parameters. Compared to the Sham group, rats in the UUO group exhibited slower body weight gain, which was ameliorated following ATG intervention (Figure 2A). Changes in survival rates similarly indicated the potential efficacy of ATG (Figure 2B). Moreover, renal morphology in the UUO group appeared abnormally swollen and pale in color, whereas other treatment groups showed varying degrees of improvement (Figure 2C). Although body weight at the time of tissue collection was significantly reduced in the UUO group compared to the Sham group (*p =* 0.002), this change was not significant in any treatment group except for the ATG + IRB group (*p =* 0.031) (Figure 2D). However, calculation of the renal index revealed a marked elevation in the UUO group (*p <* 0.001), which was significantly attenuated in all treatment groups (*p <* 0.01) (Figure 2E). Histopathological examination via HE and Masson staining of renal tissues (Figure 2F) demonstrated severe tubular dilation, epithelial cell detachment, loss of nephrons, cast formation, and inflammatory cell infiltration in the UUO group. The histopathological score based on HE staining was significantly higher in the UUO group (*p <* 0.001), while all treatment groups showed structural improvement and significantly reduced scores (*p <* 0.001) (Figure 2G). Additionally, the area of collagen fibers and collagen volume fraction were markedly increased in the UUO group (*p <* 0.001) but decreased across all treatment groups (*p <* 0.001) (Figure 2H). At the molecular level, mRNA expression of fibrosis and epithelial-mesenchymal transition (EMT)-related markers, including α-SMA (*p =* 0.002), collagen I (*p =* 0.002), and fibronectin (*p <* 0.001), was significantly upregulated in the UUO group compared to the Sham group, confirming successful induction of RF. All treatment groups significantly suppressed the expression of these factors (*p <* 0.05) (Figures 2I–K). Consistent with these findings, Western blot analysis (Figure 2L) revealed that ATG treatment also significantly downregulated the protein levels of α-SMA (*p =* 0.004), Collagen I (*p =* 0.004), and Vimentin (*p =* 0.018) (Figures 2M–O). Collectively, these results demonstrate that ATG possesses considerable therapeutic potential in ameliorating RF.

#### Transcriptomic analysis

**FIGURE 3:**
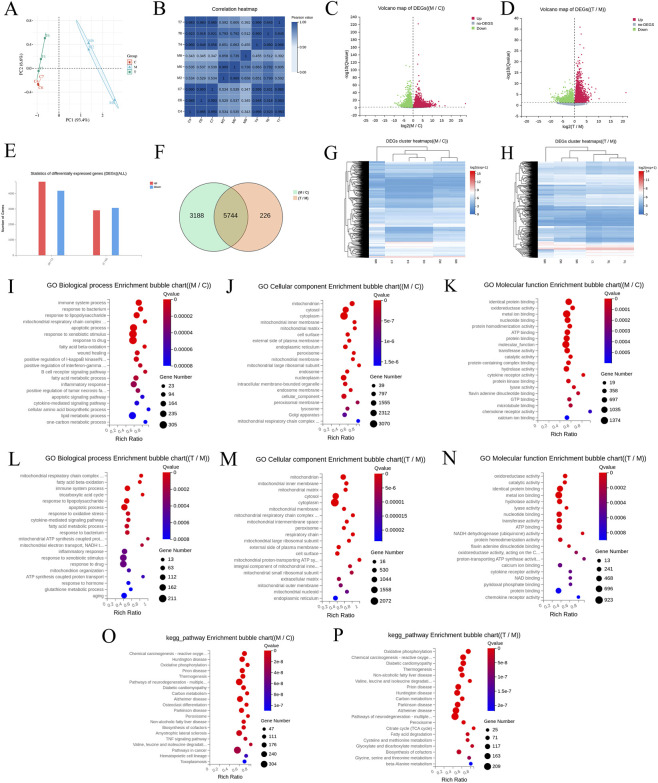
Transcriptomic analysis of the Sham group (C), UUO group (M), and ATG group (T). **(A)** Principal component analysis of all samples. **(B)** Heatmap of Pearson correlation analysis among samples. **(C,D)** Screening of differentially expressed genes (DEGs) visualized by volcano plots. **(E)** Statistical analysis of up- and downregulated DEGs. **(F)** Venn diagram illustrating the intersection of DEGs across comparisons. **(G,H)** Cluster analysis of DEGs displayed as heatmaps. **(I–K)** GO enrichment analysis for the M/C comparison group. **(L–N)** GO enrichment analysis for the T/M comparison group. **(O,P)** Identification of key pathways via KEGG enrichment analysis for the respective comparison groups.

**FIGURE 4:**
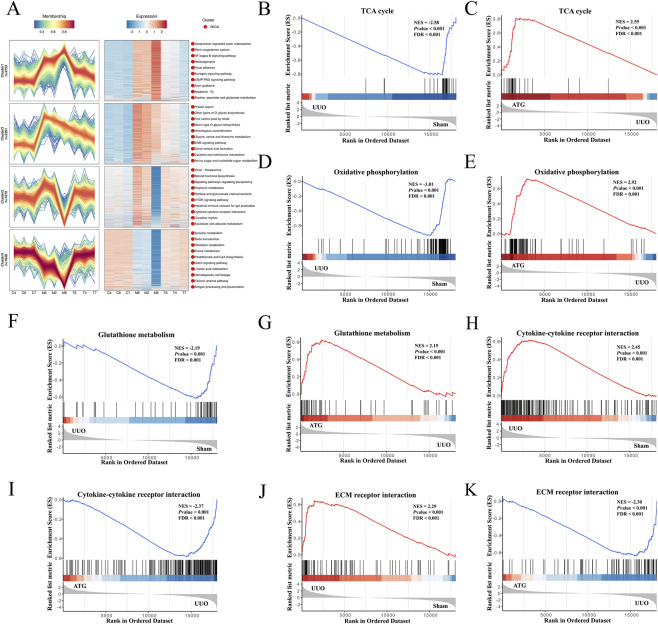
Expression pattern clustering and GSEA further elucidate the mechanism by which ATG ameliorates RF. **(A)** Expression pattern clustering analysis of genes. **(B,C)** GSEA enrichment plot for the TCA cycle pathway. **(D,E)** GSEA enrichment plot for the oxidative phosphorylation pathway. **(F,G)** GSEA enrichment plot for the glutathione metabolism pathway. **(H,I)** GSEA enrichment plot for the cytokine-cytokine receptor interaction pathway. **(J,K)** GSEA enrichment plot for the ECM-receptor interaction pathway.

To further elucidate the mechanism by which ATG ameliorates RF, we employed transcriptomic profiling to systematically identify potential key genes at the transcriptional level. For enhanced readability and presentation, group designations in the omics analysis section are represented by single letters: Sham (C), UUO (M), and ATG (T). As shown in Figure 3A, principal component analysis demonstrated good intra-group reproducibility, with the T group clustering closer to the C group compared to the M group. Pearson correlation analysis further indicated strong correlation between the T and C groups, while correlation with the M group was relatively weak (Figure 3B). Using M/C and T/M as comparative groups, post-screening scatter plots revealed downregulated genes in green and upregulated genes in red (Figures 3C,D), with statistical summaries provided in Figure 3E. Intersection analysis of the 8932 genes from the M/C group and 5970 genes from the T/M group identified 5744 key genes (Figure 3F). Cluster analysis of these genes illustrated distinct expression patterns across groups (Figures 3G,H). GO enrichment analysis indicated predominant involvement in ATP synthesis, NAD/NADH metabolism, mitochondrial function, glutathione metabolism, cytokine activity, inflammatory response, regulation of oxidative stress, NF-κB modulation, ECM organization, wound healing, and response to drugs (Figures 3I–N). KEGG pathway analysis highlighted enrichment in the TCA cycle, oxidative phosphorylation, ROS pathways, and TNF signaling (Figures 3O,P). Expression pattern clustering of key genes across the C to M to T transition revealed clusters 1-3 exhibiting an initial increase followed by a decrease, while cluster 4 showed the opposite trend. Additionally, significant enrichment was observed for the NF-κB signaling pathway and cytokine-cytokine receptor interactions (Figure 4A). GSEA further corroborated that these transcriptional changes were significantly associated with energy metabolism, inflammation, oxidative stress, and fibrotic mechanisms (Figures 4B–K). Collectively, these findings suggest that the therapeutic mechanism of ATG in ameliorating RF is closely linked to modulation of the TCA cycle, oxidative phosphorylation, inflammation, oxidative stress, and EMT.

#### Proteomic analysis

**FIGURE 5:**
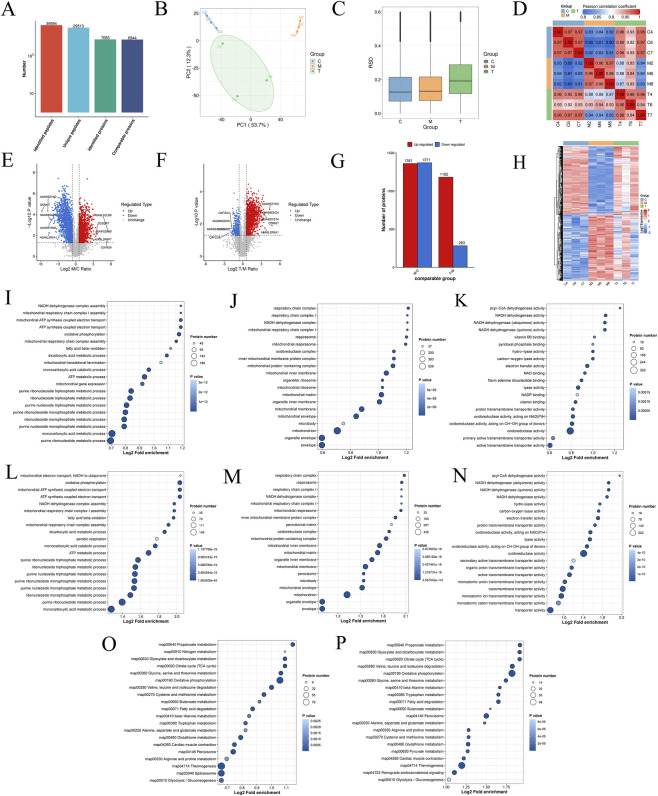
Proteomic analysis of the Sham group (C), UUO group (M), and ATG group (T). **(A)** Number of identified peptides and proteins. **(B)** Principal component analysis of all samples. **(C)** Box plot of relative standard deviations of relative quantitative values across sample groups. **(D)** Heatmap of Pearson correlation analysis among samples. **(E)** Screening of differentially expressed proteins (DEPs) in the M/C comparison group visualized by volcano plot. **(F)** Screening of DEPs in the T/M comparison group visualized by volcano plot. **(G)** Statistical analysis of up- and downregulated DEPs. **(H)** Cluster analysis of DEPs displayed as a heatmap. **(I–K)** Gene Ontology (GO) enrichment analysis for the M/C comparison group. **(L–N)** GO enrichment analysis for the T/M comparison group. **(O)** Identification of key pathways via KEGG enrichment analysis for the M/C comparison group. **(P)** Identification of key pathways via KEGG enrichment analysis for the T/M comparison group.

**FIGURE 6:**
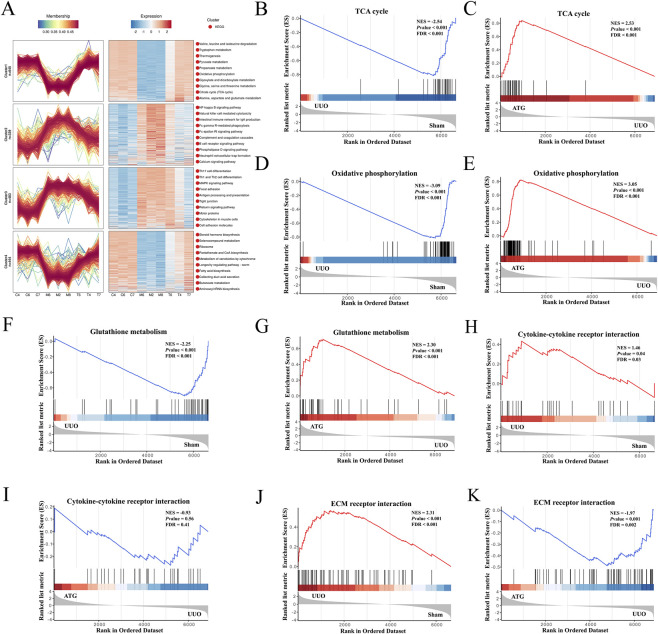
Expression pattern clustering and GSEA of proteins further elucidate the mechanism by which ATG ameliorates RF. **(A)** Expression pattern clustering analysis of proteins. **(B,C)** GSEA enrichment plot for the TCA cycle pathway. **(D,E)** GSEA enrichment plot for the oxidative phosphorylation pathway. **(F,G)** GSEA enrichment plot for the glutathione metabolism pathway. **(H,I)** GSEA enrichment plot for the cytokine-cytokine receptor interaction pathway. **(J,K)** GSEA enrichment plot for the ECM-receptor interaction pathway.

The central dogma of molecular biology indicates that genes are transcribed and subsequently translated into proteins, which serve as the primary executors of biological functions. Therefore, following transcriptomic profiling at the genetic level, proteomic analysis was conducted to further validate the underlying mechanisms. As depicted in Figure 5A, the numbers of identified peptides and proteins are summarized. Principal component analysis (Figure 5B), relative standard deviation (Figure 5C), and Pearson correlation analysis (Figure 5D) collectively demonstrated low intra-group variability, with the T group clustering closer to the C group rather than the M group. Differentially expressed proteins were screened and visualized using volcano plots (Figures 5E,F). In the M/C comparison, 1361 proteins were upregulated and 1371 downregulated, while the T/M comparison revealed 1182 upregulated and 283 downregulated proteins (Figure 5G). Cluster analysis of these differentially expressed proteins clearly segregated them into distinct upregulation and downregulation patterns, with pronounced variations across groups (Figure 5H). GO enrichment analysis highlighted predominant involvement in oxidative phosphorylation, ATP synthesis, acetyl-CoA metabolism, NAD/NADH-related processes, mitochondrial function, and redox activity (Figures 5I–N). KEGG pathway analysis further indicated significant enrichment in the TCA cycle and oxidative phosphorylation pathways (Figures 5O,P). Expression pattern clustering of key proteins revealed clusters 1 and 4 exhibiting an initial decrease followed by an increase, whereas clusters 2 and 3 showed the opposite trend. These clusters were notably associated with the NF-κB signaling pathway, TCA cycle, and oxidative phosphorylation (Figure 6A). Additionally, GSEA corroborated that alterations in key proteins were significantly linked to energy metabolism, inflammation, oxidative stress, and EMT (Figures 6B–K). These findings collectively demonstrate that, at the protein level, the mechanism by which ATG ameliorates RF is closely associated with modulation of the TCA cycle, oxidative phosphorylation, and NF-κB signaling pathway, thereby reinforcing the consistency with transcriptomic results.

### Integrated analysis of transcriptomics and proteomics

**FIGURE 7:**
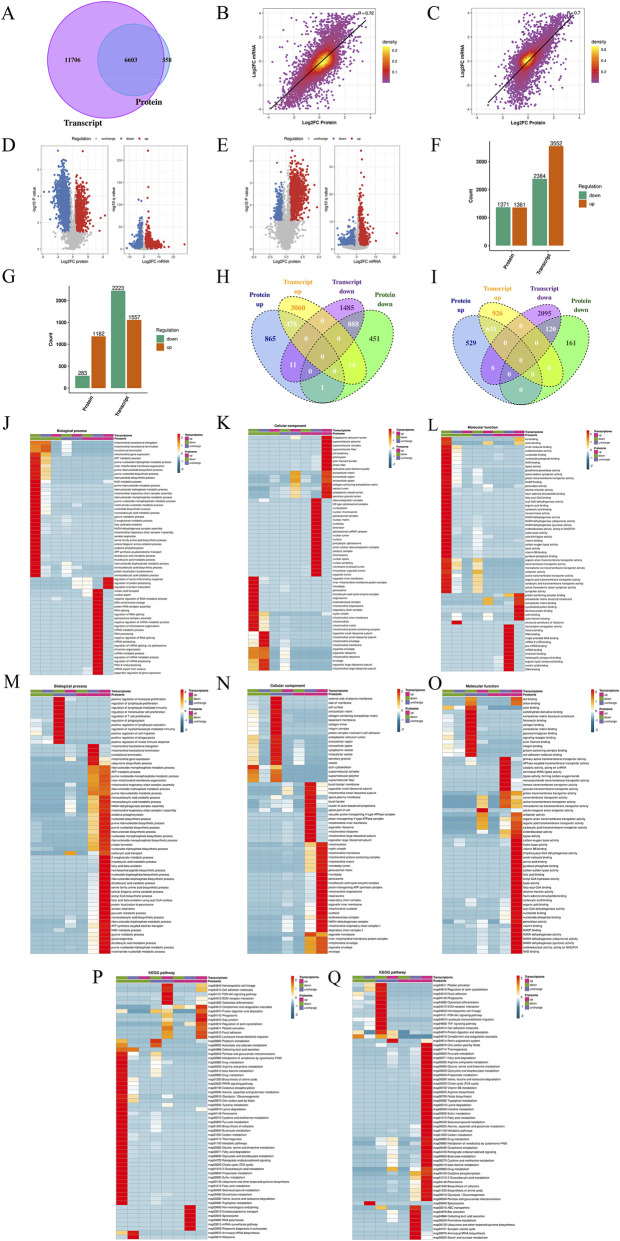
Integrated transcriptomic and proteomic analysis of the Sham group (C), UUO group (M), and ATG group (T). **(A)** Intersection of quantifiable transcripts and proteins presented as a Venn diagram. **(B)** Scatter plot and correlation analysis of transcript *versus* protein expression levels in the M/C comparison group. **(C)** Scatter plot and correlation analysis of transcript *versus* protein expression levels in the T/M comparison group. **(D)** Screening of differentially expressed genes and proteins in the M/C group visualized by volcano plot. **(E)** Screening of differentially expressed genes and proteins in the T/M group visualized by volcano plot. **(F)** Statistical analysis of up- and downregulated differentially expressed genes and proteins in the M/C group. **(G)** Statistical analysis of up- and downregulated differentially expressed genes and proteins in the T/M group. **(H)** Intersection of up- and downregulated differentially expressed genes and proteins in the M/C group displayed as a Venn diagram. **(I)** Intersection of up- and downregulated differentially expressed genes and proteins in the T/M group displayed as a Venn diagram. **(J–L)** GO enrichment analysis for the M/C comparison group. **(M–O)** GO enrichment analysis for the T/M comparison group. **(P)** Identification of key pathways via KEGG enrichment analysis for the M/C comparison group. **(Q)** Identification of key pathways via KEGG enrichment analysis for the T/M comparison group.

**FIGURE 8:**
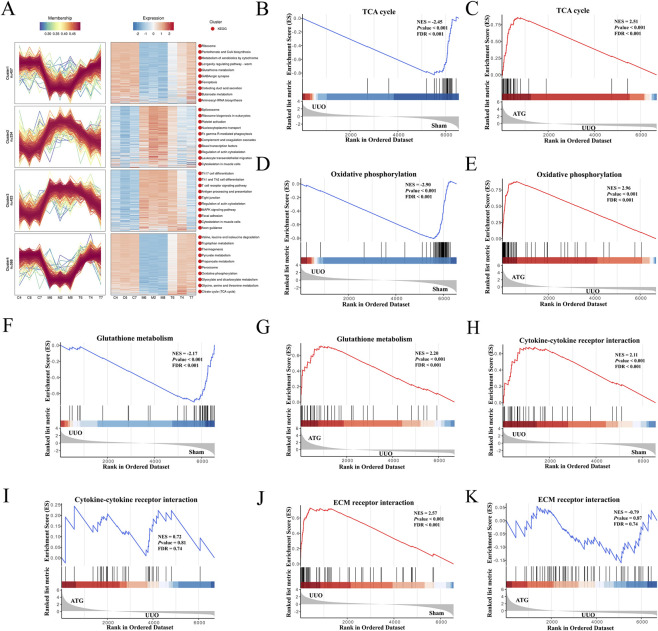
Expression pattern clustering and GSEA of genes and proteins further elucidate the mechanism by which ATG ameliorates RF. **(A)** Expression pattern clustering analysis of genes and proteins. **(B,C)** GSEA enrichment plot for the TCA cycle pathway. **(D,E)** GSEA enrichment plot for the oxidative phosphorylation pathway. **(F,G)** GSEA enrichment plot for the glutathione metabolism pathway. **(H,I)** GSEA enrichment plot for the cytokine-cytokine receptor interaction pathway. **(J,K)** GSEA enrichment plot for the ECM-receptor interaction pathway.

The aforementioned results demonstrate a significant concordance between transcriptomic and proteomic data; however, analysis of individual omics layers alone may be insufficient to fully elucidate the mechanism of drug action. To gain deeper insights into how ATG ameliorates RF, we conducted an integrated analysis of both transcriptomic and proteomic profiles. As shown in Figure 7A, intersection analysis of 18,309 transcripts and 6,961 proteins identified 6,603 key targets. Correlation analysis revealed Pearson correlation coefficients of *r* = 0.72 for the M/C comparison and *r* = 0.70 for the T/M comparison (Figures 7B,C), indicating moderate to strong concordance between transcript and protein expression changes. The identification of discordant targets—where transcript and protein levels changed in opposite directions—suggests the involvement of post-transcriptional regulatory mechanisms that may be biologically informative. For instance, transcripts that were upregulated but correspondingly downregulated at the protein level could reflect increased protein degradation or translational repression, while proteins that increased despite reduced transcript levels might indicate enhanced translation efficiency or prolonged protein stability. Such discordant features may represent key regulatory nodes that are not captured by transcriptomic analysis alone and could play important roles in the pathogenesis of RF and its modulation by ATG. Further screening visualized the number and distribution of up- and downregulated targets using volcano plots and bar charts (Figures 7D–G). Venn diagrams were employed to illustrate the overlap between differentially expressed genes and proteins (Figures 7H,I). In the M/C comparison, 478 targets showed concurrent upregulation at both transcript and protein levels, 888 were downregulated at both levels, 14 were upregulated in transcripts but downregulated in proteins, and 11 were downregulated in transcripts but upregulated in proteins. For the T/M comparison, 631 targets were upregulated at both levels, 120 were downregulated, and 8 exhibited downregulated transcripts with upregulated proteins. GO enrichment analysis highlighted involvement in oxidative phosphorylation, ATP synthesis, NAD(P)/NAD(P)H metabolism, acetyl-CoA metabolism, mitochondrial function, inflammatory response, redox regulation, cell migration, ECM organization, collagen formation, and fibronectin binding (Figures 7J–O). KEGG pathway analysis indicated significant enrichment in the TCA cycle, oxidative phosphorylation, glutathione metabolism, ECM-receptor interaction, and TNF signaling pathways (Figures 7P,Q). Expression pattern clustering of key targets (Figure 8A) revealed that clusters 1 and 4 exhibited an initial decrease followed by an increase, while clusters 2 and 3 showed the opposite trend. These clusters were predominantly associated with the TCA cycle and oxidative phosphorylation. GSEA further confirmed that these key targets were significantly linked to critical pathways involved in energy metabolism and cellular stress responses (Figures 8B–K).

Subsequently, we ranked the top 15 genes and proteins based on fold change. In the UUO vs. Sham comparison (Table 2), S100A8 ranked highest and was also identified in the ATG vs. UUO comparison (Table 3), suggesting its role as a key target in ATG-mediated amelioration of RF. Similarly, S100A9, which forms a heterodimer with S100A8, was also identified as a critical target. Existing evidence indicates that S100A8/A9 regulates ROS production via NOX, thereby influencing the NF-κB pathway (Ghavami et al., 2010; Vlad et al., 2025). Consistent with this, our multi-omics data showed significant enrichment in NF-κB signaling, TCA cycle, oxidative phosphorylation, inflammation, oxidative stress, and EMT. Based on these findings, we propose that ATG may alleviate RF through the S100A8/A9/NOX/NF-κB signaling axis, modulating TCA cycle and oxidative phosphorylation, thereby suppressing inflammation and oxidative stress-driven EMT.

**TABLE 2:** Top 15 genes/Proteins ranked by fold change (UUO vs. Sham) from integrated transcriptomic and proteomic analysis.

Transcription ID	Protein accession	log2fold change (protein)	log10p value (protein)	log2fold change (gene)	log10p value (gene)
S100a8	P50115	6.38	2.54	10.54	6.17
Np4	A0A8L2QLB0	5.53	2.35	4.23	0.40
Camp	G3V8S9	5.31	1.38	5.21	0.96
Kng1	A0A8L2R8P7	4.02	1.73	3.32	2.28
S100a14	A0A8I6A7N5	3.65	1.15	2.82	6.40
Sprr1a	A0A8I5ZKS9	3.56	1.25	9.52	9.70
Plod2	D3ZQR7	3.55	2.19	3.59	6.88
Kng2	A0A8I5ZK39	3.38	1.92	−0.01	0.004
Lgals3bp	A0A8I6A7P6	3.33	2.72	2.89	12.17
RT1-DMa	A0A8I6AFU3	3.27	4.01	1.55	3.66
Krt6a	A0A8I6G380	3.26	1.05	11.74	10.70
Eln	A0A0G2JST5	3.25	2.13	4.83	11.30
Krt14	Q6IFV1	3.05	1.55	9.68	15.98
Rbm3	Q925G0	3.05	3.53	0.78	1.04
Slc34a2	A0A0H2UHZ0	3.03	2.42	3.51	2.94

**TABLE 3:** Top 15 genes/Proteins ranked by fold change (ATG vs. UUO) from integrated transcriptomic and proteomic analysis.

Transcription ID	Protein accession	log_2_fold change (protein)	log_10_ *p* value (protein)	log_2_fold change (gene)	log_10_ *p* value (gene)
Krt15	Q6IFV3	−3.12	0.65	−3.65	2.59
Krt6a	A0A8I6G380	−3.07	0.96	−6.75	5.05
Ngp	D3ZY96	−2.86	1.02	−4.03	0.84
Camp	G3V8S9	−2.66	0.89	−4.61	0.90
Mpo	A0A0G2K1A2	−2.64	0.90	−4.05	0.79
S100a9	A0A0H2UHJ1	−2.58	0.70	−6.70	3.38
Krt5	Q6P6Q2	−2.56	0.57	−3.37	4.58
Cd177	M0R8W9	−2.51	0.89	−1.93	0.24
Ctsg	A0A8I5XFF7	−2.48	0.77	−2.99	0.20
Np4	A0A8L2QLB0	−2.46	1.03	−4.48	0.38
Elane	D4A488	−2.45	0.67	−3.17	0.66
Anxa8	Q4FZU6	−2.36	1.40	−3.76	8.16
Krt14	Q6IFV1	−2.33	1.14	−3.92	10.62
S100a14	A0A8I6A7N5	−2.19	0.86	−2.05	2.51
S100a8	P50115	−2.18	0.85	−6.81	3.35

#### ATG alleviates RF via regulation of the S100A8/A9/NOX/NF-κB signaling pathway

**FIGURE 9:**
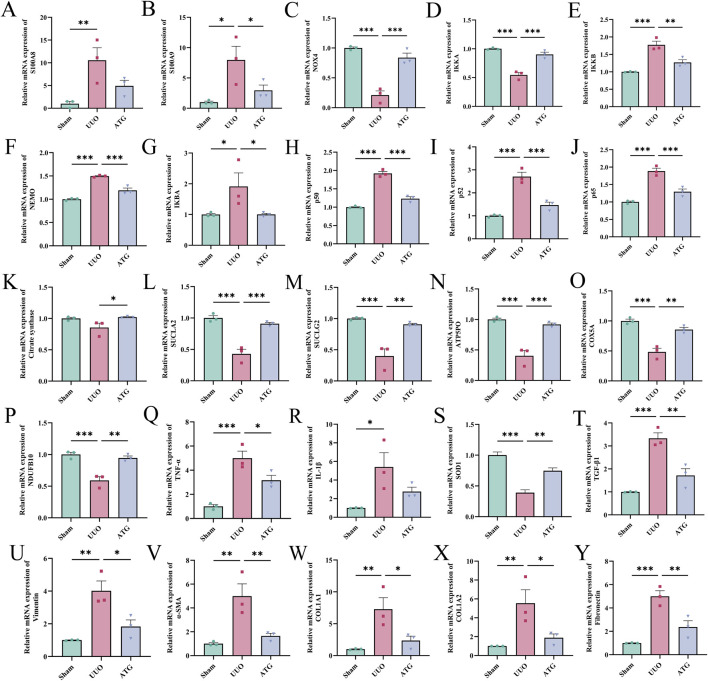
Relative mRNA expression levels of key differentially expressed genes associated with ATG-mediated amelioration of RF across experimental groups. **(A)** S100A8. **(B)** S100A9. **(C)** NOX4. **(D)** IKKA. **(E)** IKKB. **(F)** NEMO. **(G)** IKBA. **(H)** p50. **(I)** p52. **(J)** p65. **(K)** Citrate synthase. **(L)** SUCLA2. **(M)** SUCLG2. **(N)** ATP5PO. **(O)** COX5A. **(P)** NDUFB10. **(Q)** TNF-α. **(R)** IL-1β. **(S)** SOD1. **(T)** TGF-β1. **(U)** Vimentin. **(V)** α-SMA. **(W)** COL1A1. **(X)** COL1A2. **(Y)** Fibronectin. Data are presented as mean ± SEM, *n* = 3 per group, *p < 0.05, **p < 0.01, ***p < 0.001.

**FIGURE 10:**
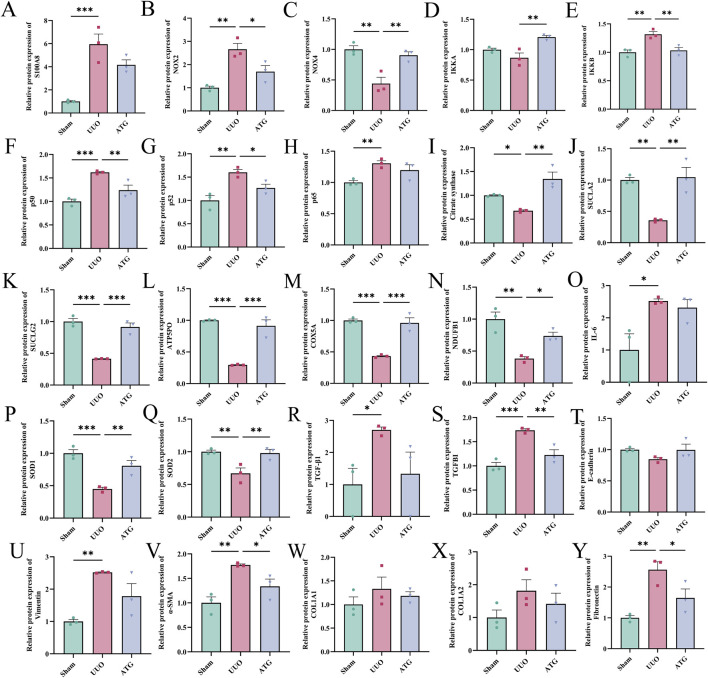
Relative protein expression levels of key differentially expressed proteins associated with ATG-mediated amelioration of RF across experimental groups. **(A)** S100A8. **(B)** NOX2. **(C)** NOX4. **(D)** IKKA. **(E)** IKKB. **(F)** p50. **(G)** p52. **(H)** p65. **(I)** Citrate synthase. **(J)** SUCLA2. **(K)** SUCLG2. **(L)** ATP5PO. **(M)** COX5A. **(N)** NDUFB1. **(O)** IL-6. **(P)** SOD1. **(Q)** SOD2. **(R)** TGF-β1. **(S)** TGFBI. **(T)** E-cadherin. **(U)** Vimentin. **(V)** α-SMA. **(W)** COL1A1. **(X)** COL1A2. **(Y)** Fibronectin. Data are presented as mean ± SEM, *n* = 3 per group, *p < 0.05, **p < 0.01, ***p < 0.001.

**FIGURE 11:**
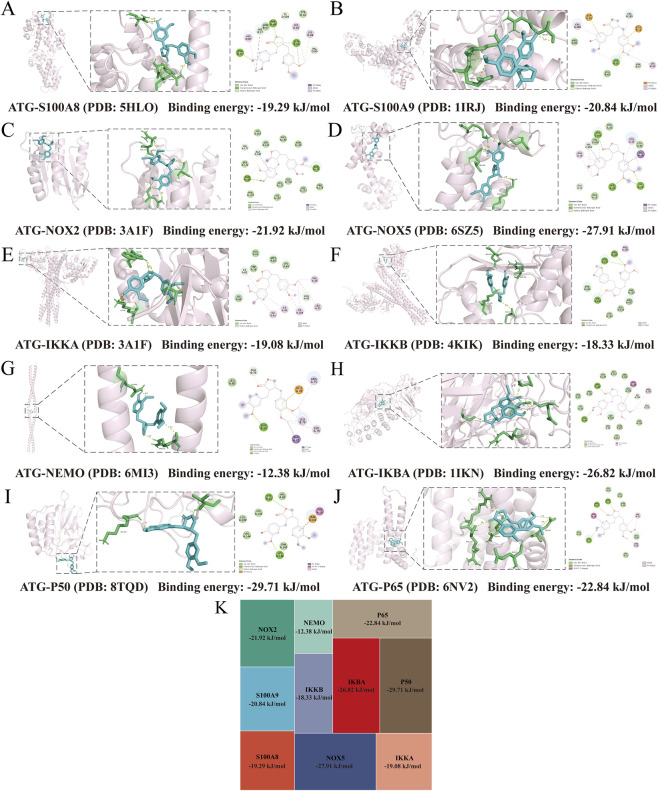
Molecular docking analysis and visualization of the binding mode and binding energy between ATG and key target proteins within the S100A8/A9/NOX/NF-κB signaling pathway. **(A)** S100A8. **(B)** S100A9. **(C)** NOX2. **(D)** NOX5. **(E)** IKKA. **(F)** IKKB. **(G)** NEMO. **(H)** IKBA. **(I)** p50. **(J)** p65. **(K)** Binding energies of molecular docking across experimental groups.

To validate this hypothesis, we initially examined key differentially expressed genes (Figure 9) and proteins (Figure 10) from our multi-omics datasets, assessing their expression levels across experimental groups. The results demonstrated that, compared to the UUO group, ATG treatment suppressed the expression of S100A8/A9, downregulated NOX2, upregulated NOX4, inhibited the NF-κB signaling axis, restored TCA cycle and oxidative phosphorylation impairments, attenuated inflammation and oxidative stress, mitigated EMT, and reduced fibrotic factor expression. Molecular docking analysis further revealed that ATG exhibited binding energies below −5 kJ/mol for key targets within the S100A8/A9/NOX/NF-κB pathway, with interactions such as hydrogen bonds and π-π stacking enhancing binding affinity, indicating strong binding potential (Figure 11).

**FIGURE 12:**
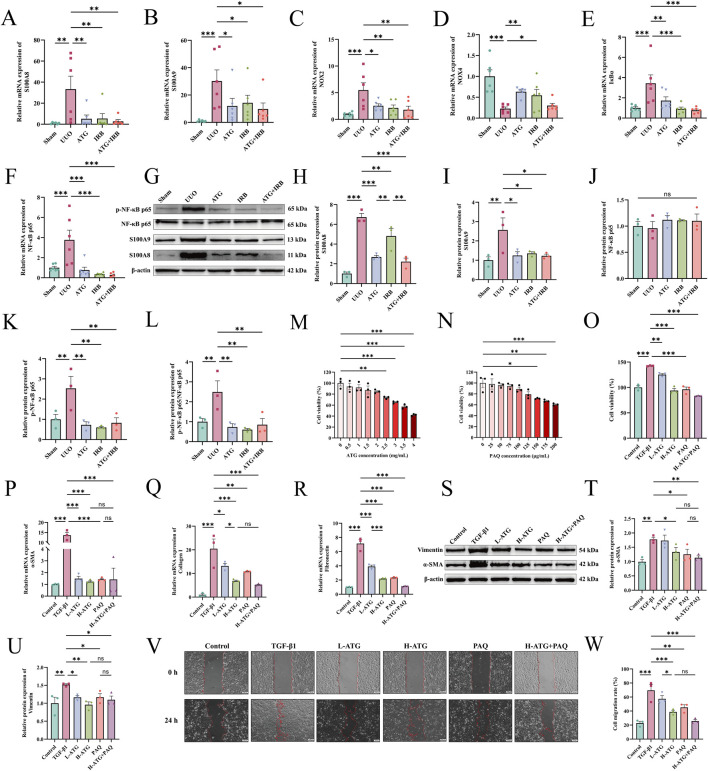
ATG ameliorates UUO-induced RF in rats by modulating the S100A8/A9/NOX/NF-κB signaling pathway, and additionally attenuates TGF-β1-induced fibrotic responses in HK-2 cells. **(A–F)** Relative mRNA expression levels of key genes (S100A8, S100A9, NOX2, NOX4, IκBα, and NF-κB p65) in the S100A8/A9/NOX/NF-κB pathway were measured via RT-qPCR in renal tissues. **(G–L)** Protein expression levels of S100A8, S100A9, NF-κB p65, and phosphorylated NF-κB p65 were detected using Western blot, including representative bands and quantitative analysis. **(M)** Cell viability of HK-2 cells under varying concentrations of ATG was assessed via CCK-8 assay. **(N)** Cell viability under different concentrations of PAQ was similarly evaluated using CCK-8. **(O)** Changes in HK-2 cell viability before and after drug treatment were determined via CCK-8. **(P-R)** mRNA expression levels of α-SMA, collagen I, and fibronectin in cells were quantified through RT-qPCR. **(S–U)** Western blot results and quantitative analysis of α-SMA and vimentin protein expression in cells. **(V–W)** Representative images of wound healing assays at 0 h and 24 h (scale bar = 100 μm) along with quantitative analysis of cell migration rates. Data are presented as mean ± SEM, *n* = 3 per group (*n* = 6 per group for A-F), *p < 0.05, **p < 0.01, ***p < 0.001, ns, no significant.

To independently validate the key targets identified through multi-omics analysis, we examined their expression levels using experimental approaches in both animal tissues and cell culture samples distinct from those used for omics profiling. In renal tissues, ATG significantly inhibited the mRNA expression of S100A8 (*p =* 0.004), S100A9 (*p =* 0.027), NOX2 (*p =* 0.01), IκBα (*p =* 0.008), and NF-κB p65 (*p <* 0.001), while upregulating NOX4 expression (*p =* 0.01) (Figures 12A–F). Similarly, at the protein level, ATG suppressed the expression of S100A8 (*p <* 0.001)/A9 (*p =* 0.013) and phosphorylation of NF-κB p65 (*p =* 0.002) (Figures 12G–L).

**FIGURE 13:**
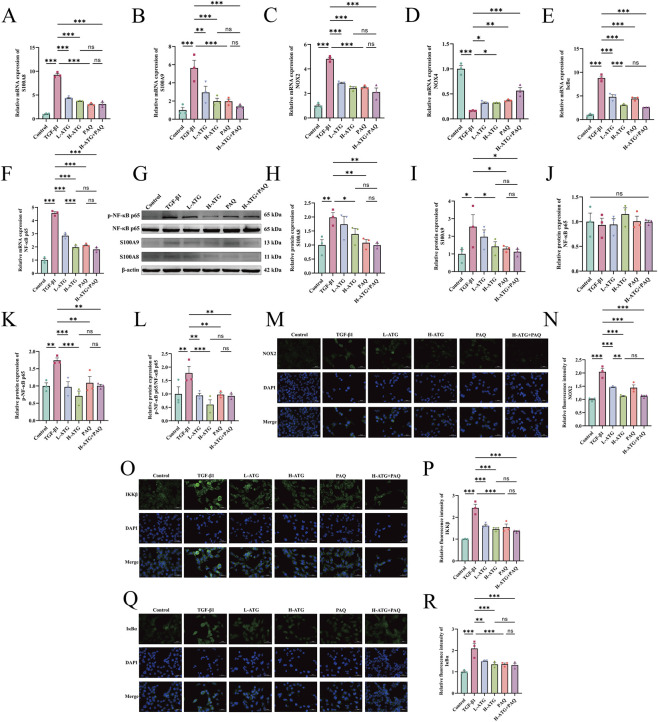
ATG alleviates TGF-β1-induced fibrosis in HK-2 cells by regulating the S100A8/A9/NOX/NF-κB signaling pathway. **(A–F)** Relative mRNA expression levels of key genes (S100A8, S100A9, NOX2, NOX4, IκBα, and NF-κB p65) in the S100A8/A9/NOX/NF-κB signaling pathway were measured in cells using RT-qPCR. **(G–L)** Relative protein expression levels of key proteins (S100A8, S100A9, NF-κB p65, and phosphorylated NF-κB p65) in the S100A8/A9/NOX/NF-κB signaling pathway were detected by Western blot, including representative band images and quantitative analysis. **(M,N)** Immunofluorescence images of NOX2 in cells and quantitative analysis of relative fluorescence intensity. **(O,P)** Immunofluorescence images of IKKβ in cells and quantitative analysis of relative fluorescence intensity. **(Q,R)** Immunofluorescence images of IκBα in cells and quantitative analysis of relative fluorescence intensity (scale bar = 50 μm). Data are presented as mean ± SEM, *n* = 3 per group, *p < 0.05, **p < 0.01, ***p < 0.001, ns, no significant.

To obtain pathway-specific functional confirmation of the involvement of the S100A8/A9 axis in ATG-mediated anti-fibrotic effects, we employed PAQ, a specific pharmacological inhibitor of S100A8/A9, in TGF-β1-induced HK-2 cells. This approach allowed us to directly assess whether blockade of this pathway could recapitulate the effects of ATG, thereby providing causal evidence for its functional relevance. Concentration-dependent assays indicated a significant reduction in cell viability starting at 2.5 mg/mL for ATG (*p =* 0.01) (Figure 12M) and 150 μg/mL for PAQ (*p =* 0.024) (Figure 12N). Consequently, ATG at 2 mg/mL and PAQ at 125 μg/mL were selected for subsequent experiments. Cell viability assays showed a significant increase in the TGF-β1 group compared to the control group (*p <* 0.001), likely due to TGF-β1-induced proliferative transformation, which was reversed by drug treatments (*p <* 0.01) (Figure 12O). RT-qPCR analysis of fibrotic factors revealed elevated mRNA levels of α-SMA, collagen I, and fibronectin in the TGF-β1 group compared to controls (*p <* 0.001). Both L-ATG (α-SMA, *p <* 0.001; Collagen I, *p =* 0.012; Fibronectin, *p <* 0.001) and H-ATG (*p <* 0.001) groups exhibited dose-dependent reductions in these factors, with similar effects observed in the PAQ (α-SMA, *p <* 0.001; Collagen I, *p =* 0.002; Fibronectin, *p <* 0.001) group. However, no significant differences were detected among the H-ATG, PAQ, and H-ATG + PAQ groups (Figures 12P–R), suggesting that ATG alone nearly maximally inhibited the S100A8 axis, leaving little additive effect for PAQ. Western blot analysis confirmed that ATG (α-SMA, *p =* 0.04; Vimentin, *p =* 0.002) and PAQ (α-SMA, *p =* 0.018; Vimentin, *p =* 0.027) suppressed the protein expression of α-SMA and vimentin (Figures 12S–U). Wound healing assays further demonstrated significantly reduced healing area and cell migration rates in the ATG (*p <* 0.001) and PAQ (*p =* 0.003) groups compared to the TGF-β1 group (Figures 12V,W). Subsequent RT-qPCR results indicated that all drug treatments significantly inhibited the mRNA expression of S100A8, S100A9, NOX2, IκBα, and NF-κB p65 (*p <* 0.01) while upregulating NOX4 (*p <* 0.05), with no significant differences observed among the H-ATG, PAQ, and H-ATG + PAQ groups (Figures 13A–F). At the protein level, both ATG (S100A8, *p =* 0.042; S100A9, *p =* 0.047; p-NF-κB p65/NF-κB p65, *p <* 0.001) and PAQ (S100A8, *p =* 0.005; S100A9, *p =* 0.031; p-NF-κB p65/NF-κB p65, *p =* 0.007) suppressed S100A8/A9 expression and NF-κB p65 phosphorylation (Figures 13G–L). Immunofluorescence analysis revealed increased relative fluorescence intensity for NOX2, IKKβ, and IκBα in the TGF-β1 group compared to controls (*p <* 0.001), which was markedly reduced following drug treatment (*p <* 0.01) (Figures 13M–R). Collectively, these findings indicate that ATG alleviates renal fibrosis by modulating the S100A8/A9/NOX/NF-κB signaling pathway in a S100A8/A9-dependent manner.

#### ATG ameliorates RF by regulating TCA cycle and oxidative phosphorylation via the S100A8 signaling axis, thereby inhibiting inflammation and oxidative stress-driven EMT

**FIGURE 14:**
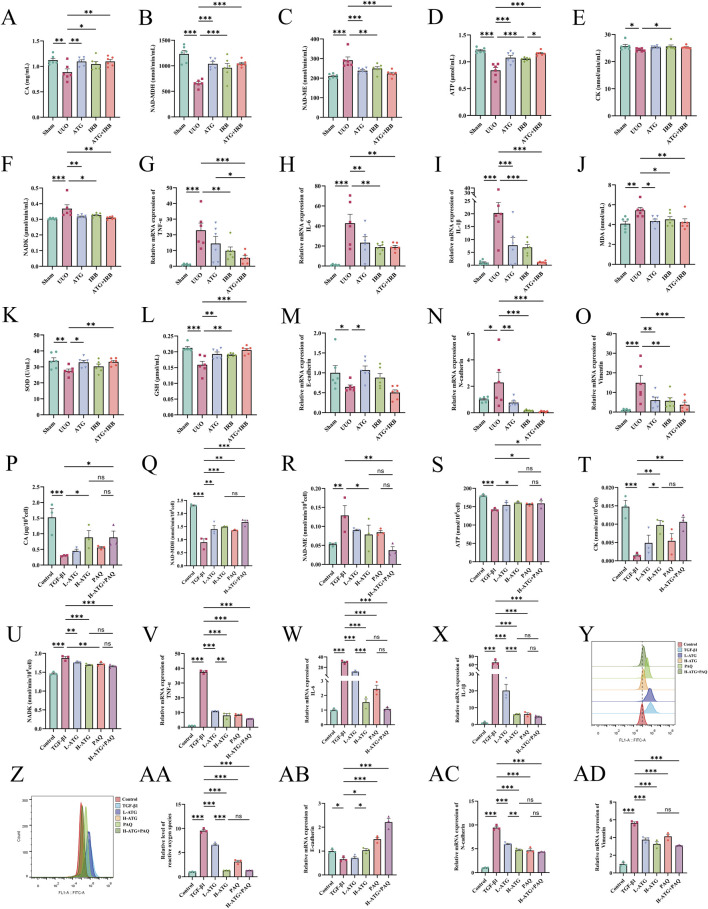
ATG ameliorates UUO-induced RF and TGF-β1-induced fibrosis in HK-2 cells by regulating the S100A8/A9/NOX/NF-κB signaling pathway, which modulates TCA cycle and oxidative phosphorylation disruptions, thereby suppressing inflammation and oxidative stress-driven EMT. **(A–C)** Expression levels of TCA cycle-related factors (CA, NAD-MDH, NAD-ME) in renal tissues. **(D–F)** Expression levels of oxidative phosphorylation-related factors (ATP, CK, NADK) in renal tissues. **(G–I)** Relative mRNA expression levels of inflammatory factors (TNF-α, IL-6, IL-1β) in renal tissues detected by RT-qPCR. **(J–L)** Expression levels of oxidative stress markers (MDA, SOD, GSH) in renal tissues. **(M–O)** Relative mRNA expression levels of EMT markers (E-cadherin, N-cadherin, Vimentin) in renal tissues detected by RT-qPCR. **(P–R)** Expression levels of TCA cycle-related factors (CA, NAD-MDH, NAD-ME) in HK-2 cells. **(S–U)** Expression levels of oxidative phosphorylation-related factors (ATP, CK, NADK) in HK-2 cells. **(V–X)** Relative mRNA expression levels of inflammatory factors (TNF-α, IL-6, IL-1β) in HK-2 cells detected by RT-qPCR. **(Y-AA)** Relative levels of ROS in HK-2 cells measured by flow cytometry. **(AB-AD)** Relative mRNA expression levels of EMT markers (E-cadherin, N-cadherin, Vimentin) in HK-2 cells detected by RT-qPCR. Data are presented as mean ± SEM, *n* = 6 per group (*n* = 3 per group for P-AD), *p < 0.05, **p < 0.01, ***p < 0.001, ns, no significant.

To investigate the relationship between the S100A8/A9/NOX/NF-κB signaling pathway and RF in the context of ATG-mediated amelioration, we further validated the downstream mechanisms of the S100A8 axis. In renal tissues, compared to the Sham group, the UUO group exhibited significant reductions in TCA cycle factors CA (*p =* 0.001) and NAD-MDH (*p <* 0.001), and an increase in NAD-ME (*p <* 0.001). All drug treatment groups reversed these aberrant changes (*p <* 0.05) (Figures 14A–C). Additionally, ATG restored the expression levels of oxidative phosphorylation-related factors ATP (*p <* 0.001), CK (*p =* 0.14), and NADK (*p =* 0.004) (Figures 14D–F). RT-qPCR analysis revealed that, compared to the UUO group, the ATG (TNF-α, *p =* 0.07; IL-6, *p =* 0.009; IL-1β, *p <* 0.001), IRB (TNF-α, *p =* 0.007; IL-6, *p =* 0.002; IL-1β, *p <* 0.001), and ATG + IRB (TNF-α, *p <* 0.001; IL-6, *p =* 0.002; IL-1β, *p <* 0.001) groups significantly suppressed the mRNA expression of inflammatory factors TNF-α, IL-6, and IL-1β (Figures 14G–I). Furthermore, drug treatments markedly reduced the oxidative stress marker MDA while elevating the antioxidant factors SOD and GSH (*p <* 0.05) (Figures 14J–L). ATG also reversed the mRNA expression levels of EMT markers E-cadherin (*p =* 0.013), N-cadherin (*p =* 0.005), and vimentin (*p =* 0.006) (Figures 14M–O). Similar mechanisms were corroborated in an *in vitro* TGF-β1-induced HK-2 cell fibrosis model. The H-ATG (CA, *p =* 0.034; NAD-MDH, *p <* 0.001; NAD-ME, *p =* 0.04) and PAQ (CA, *p =* 0.306; NAD-MDH, *p =* 0.003; NAD-ME, *p =* 0.065) groups significantly upregulated CA and NAD-MDH levels while downregulating NAD-ME, though the L-ATG (CA, *p =* 0.544; NAD-MDH, *p =* 0.002; NAD-ME, *p =* 0.102) group showed limited efficacy (Figures 14P–R). ATG also modulated ATP (*p =* 0.013), CK (*p =* 0.003), and NADK (*p <* 0.001) levels to alleviate oxidative phosphorylation impairment (Figures 14S–U). Regarding cellular inflammation, both ATG and PAQ inhibited the expression of TNF-α, IL-6, and IL-1β (*p <* 0.001) (Figures 14V–X). Flow cytometry analysis demonstrated that ATG and PAQ significantly reduced ROS levels, particularly in the H-ATG and H-ATG + PAQ groups (*p <* 0.001) (Figures 14A–Y). Finally, RT-qPCR results indicated that all drug treatments upregulated E-cadherin and downregulated N-cadherin and vimentin compared to the UUO group (*p <* 0.05) (Figures 14AB–AD). The observation that PAQ treatment alone mimicked the anti-fibrotic effects of ATG, and that combined treatment with ATG and PAQ did not produce additive effects, provides direct functional evidence that ATG acts predominantly through the S100A8/A9 signaling axis. These findings move beyond correlative associations to establish pathway-specific functional confirmation. In summary, these findings confirm that ATG alleviates RF by modulating TCA cycle and oxidative phosphorylation via the S100A8 axis, thereby suppressing inflammation and oxidative stress-driven EMT.

## Discussion

RF represents a challenging and largely irreversible process wherein renal function progressively deteriorates from a healthy state to injury, further advancing to functional loss (Dalal et al., 2025; Singh et al., 2026). During this progression, the gradual decline in function of renal tubules and glomeruli leads to parenchymal pathological changes, potentially culminating in renal failure (Niculae et al., 2023). Clinically, patients with RF often present with symptoms including edema, elevated blood pressure, loss of appetite, anemia, nausea, vomiting, and pruritus. These manifestations not only severely impact patients’ quality of life but may also precipitate additional complications such as cardiovascular diseases and osteoporosis, thereby exacerbating overall health risks (Panizo et al., 2021). From a pathological perspective, RF does not uniformly affect the entire renal parenchyma but rather initiates locally, forming distinct fibrotic niches. These niches establish a specific tissue microenvironment conducive to excessive deposition of ECM and the formation of scar tissue (Bülow and Boor, 2019). The development of these fibrotic niches is accompanied by activation of the EMT process, along with progressive accumulation and expansion of ECM, leading to lesion formation and ultimately resulting in fibrosis of the entire tissue and organ. In recent years, exploring effective treatments for RF from TCM has become a focal point of research (Yang et al., 2025). *Fructus arctii*, the dried mature fruit of *A. lappa* L. (Asteraceae), is a TCM herb first documented in the classical medical text *Mingyi Bielu*. Its direct active component, ATG, exhibits multiple pharmacological effects including anti-inflammatory, anti-fibrotic, and antioxidant properties, as well as potential in treating diabetic nephropathy (Wang et al., 2023). Based on this evidence, we hypothesize that ATG represents a highly promising candidate for inhibiting RF. However, the therapeutic efficacy and underlying mechanisms of ATG in ameliorating RF remain unclear, particularly from a multi-omics perspective.

Against this backdrop, we comprehensively investigated the therapeutic effects and mechanisms of ATG in improving RF using a multi-omics approach. Initially, by assessing renal function and fibrotic marker levels in a rat model, we demonstrated that ATG exerts a dose-dependent improvement in RF. The high-dose ATG group showed effects comparable to or exceeding those of the positive control group (IRB), with significant differences compared to the UUO group. Consequently, the high-dose ATG group was selected for subsequent experimental validation. Through a series of pharmacodynamic experiments, we further confirmed that ATG ameliorates UUO-induced RF in rats, as evidenced by improvements in renal morphology, functional parameters, and fibrotic markers at both genetic and protein levels. Unlike conventional studies, we included a combination therapy group (ATG + IRB), which exhibited non-significant trends toward enhanced efficacy compared to either monotherapy alone. While these observations hint at potential additive or complementary effects, the lack of statistical significance and the absence of formal synergy analysis preclude definitive conclusions regarding true synergism. Nonetheless, these trends provide a preliminary rationale for future studies designed to rigorously evaluate combination strategies in RF treatment. Similarly, *in vitro* experiments demonstrated that ATG alleviates TGF-β1-induced fibrosis and migration in HK-2 cells.

The central dogma of molecular biology underscores the flow of genetic information from DNA to RNA to proteins. Accordingly, elucidating drug mechanisms requires analysis from both genetic and proteomic perspectives. Aligning with contemporary research trends (Eddy et al., 2020), we employed transcriptomic and proteomic analyses, along with integrated multi-omics approaches, to delve into the mechanisms underlying ATG’s effects on RF. Following identification of differentially expressed genes and proteins, enrichment analysis revealed associations with the NF-κB signaling pathway, TCA cycle, oxidative phosphorylation, inflammation, oxidative stress, EMT, and ECM regulation. Further screening identified S100A8 and S100A9 among the top 15 differentially expressed genes/proteins in the integrated omics analysis, suggesting their potential centrality in ATG’s mechanism of action.

S100A8, a calcium-binding protein of the S100 family, is abundant in neutrophil cytoplasm. Under healthy renal conditions, S100A8 expression is relatively low, primarily localized in neutrophils, monocytes, macrophages, and keratinocytes. However, its expression is significantly upregulated during RF, implicating it as a key player in fibrotic progression. Studies indicate that S100A8 frequently functions as a heterodimer with S100A9 (Chen et al., 2023). Consistent with this, our analysis revealed parallel expression trends for S100A9, reinforcing our hypothesis. Research demonstrates that aberrant expression of S100A8/A9 under pathological conditions exacerbates RF by promoting EMT (Du et al., 2023). Moreover, inhibiting S100A8/A9 alleviates tubular epithelial cell apoptosis, inflammation, and superoxide production, thereby improving septic nephropathy (Shi et al., 2023). S100A8/A9 also promotes endometrial fibrosis via the RAGE/JAK2/STAT3 signaling pathway (Xin et al., 2024). Thus, S100A8/A9 may represent a critical therapeutic target and novel strategy for RF. Our findings further indicated significantly elevated NOX protein expression in the model group, which was reduced following ATG treatment. Existing literature reports that S100A8/A9 promotes NOX activation through arachidonic acid and calcium pathways (Kerkhoff et al., 2005), regulates intracellular calcium concentrations to activate NOX (Schenten et al., 2010), and activates NOX via neutrophil cytosolic factor-1 (van Dalen et al., 2018). Multiple studies have established that NOX activation exacerbates oxidative stress by promoting ROS production (Huang Y. F. et al., 2023; Pecchillo Cimmino et al., 2023; Vermot et al., 2021). ROS generation and alterations in redox status influence the NF-κB signaling pathway by affecting IKK activation, IκB activation and ubiquitin-proteasome degradation, NF-κB phosphorylation, and nuclear translocation, thereby promoting transcription of downstream genes (Morgan and Liu, 2011). Previous research indicates that the NF-κB pathway regulates the TCA cycle and oxidative phosphorylation, potentially involving activation of NAD-ME and NADK transcription and inhibition of NAD-MDH and CK transcription (Li L. et al., 2024; Zhou et al., 2017). Suppression of the TCA cycle and oxidative phosphorylation—as manifestations of energy metabolism dysfunction—triggers activation of inflammation (TNF-α, IL-6, IL-1β) and oxidative stress (MDA), while inhibiting antioxidant responses (SOD, GSH) (Li R. et al., 2024; MacLean et al., 2023). This cascade activates EMT (E-cadherin, N-cadherin, vimentin) and increases ECM deposition (α-SMA, Collagen I, vimentin), ultimately aggravating RF (Lovisa et al., 2015).

Based on these findings and our results, we propose that ATG may ameliorate RF by modulating the S100A8/A9/NOX/NF-κB signaling axis, thereby regulating impairments in the TCA cycle and oxidative phosphorylation, and subsequently inhibiting inflammation and oxidative stress-driven EMT. An important strength of this study is the independent experimental validation of key targets identified through multi-omics analysis. The differential expression of S100A8, S100A9, and downstream signaling molecules was confirmed using RT-qPCR and Western blot in renal tissues from UUO rats, as well as in TGF-β1-induced HK-2 cells. These validation experiments, performed on biological samples independent from those used for omics profiling, corroborate the reliability of the multi-omics findings. Furthermore, the functional consequences of S100A8/A9 pathway modulation were assessed through measurements of inflammatory cytokines, oxidative stress markers, TCA cycle intermediates, and oxidative phosphorylation parameters, providing multi-dimensional experimental evidence supporting the proposed mechanism. Together, these independent validation experiments bridge the gap between omics-driven hypothesis generation and mechanistic confirmation. Notably, we introduced PAQ, a specific S100A8/A9 inhibitor, to provide pathway-specific functional confirmation of the proposed mechanism. While multi-omics analysis revealed correlative associations between S100A8/A9 dysregulation and ATG treatment, the PAQ experiments established a causal relationship. PAQ treatment alone recapitulated the effects of ATG, including suppression of fibrotic markers, inhibition of NF-κB phosphorylation, attenuation of inflammatory and oxidative stress responses, and restoration of TCA cycle and oxidative phosphorylation function. Furthermore, the lack of additive effects when ATG and PAQ were combined indicates that ATG exerts its therapeutic effects primarily through modulation of the S100A8/A9 axis. These findings transform the initial correlative observations into functionally validated mechanistic insights, addressing the need for pathway-specific confirmation. While most results aligned with our hypotheses, several observations were unexpected. In our study, an intriguing observation was the inverse correlation between NOX2 and NOX4 expression following ATG treatment: NOX2 was downregulated, whereas NOX4 was upregulated. This finding may initially appear counterintuitive, as NOX4 has been widely reported to promote fibrosis in various models of CKD (Zhang C. et al., 2025; Zho et al., 2025). However, a growing body of evidence suggests that the role of NOX4 is context-dependent and far more complex than originally appreciated. NOX4 has been implicated in cellular signaling, stress adaptation, and the maintenance of cellular homeostasis. Notably, several studies have demonstrated that NOX4 deficiency exacerbates, rather than ameliorates, RF in certain models. Nlandu Khodo et al. (Nlandu Khodo et al., 2012) reported that NOX4 knockout mice developed more severe RF following chronic injury, suggesting a protective role for NOX4 in the kidney. Similarly, the existing literature on the role of NOX4 in the kidney presents conflicting findings; experiments using NOX4-deficient mice have indicated that NOX4 exerts a protective effect on renal tubular cells and plays a role in metabolic regulation upon injury, with systemic NOX4 deletion rendering mice more susceptible to acute and chronic tubular damage (Rajaram et al., 2019). These protective effects may be attributed to NOX4-mediated modulation of redox signaling pathways that promote cellular adaptation and survival. In the context of our study, the upregulation of NOX4 following ATG treatment may therefore represent a compensatory or adaptive response that contributes to the resolution of fibrosis, rather than a pro-fibrotic signal. This interpretation is consistent with the concurrent downregulation of inflammatory cytokines and fibrotic factors observed in the treatment groups. Secondly, although IκBα is typically degraded via the ubiquitin-proteasome pathway upon activation, its levels were elevated in the UUO group with activated NF-κB signaling but reduced in the ATG group. Prior research suggests this may reflect rapid negative feedback replenishment of IκBα upon NF-κB pathway activation (Wang et al., 2020). Regarding key factors downstream of NF-κB regulating energy metabolism: CK reversibly catalyzes the transphosphorylation between creatine and ATP, playing a vital role in energy turnover and ATP regeneration (Kreider and Stout, 2021)—hence its increase in the ATG group. NAD-ME reduces NAD^+^ to NADH, NADK converts NAD^+^ to NADP^+^, and NAD-MDH catalyzes the interconversion of NAD^+^ and NADH. Depletion of NAD^+^ exacerbates kidney disease (Morevati et al., 2022), likely explaining the reduction in NAD-ME and NADK, and the increase in NAD-MDH following ATG treatment.

Beyond its preclinical efficacy, ATG possesses several attributes that support its clinical translational potential. First, as the active component of *F. arctii*—a widely used medicinal and edible plant with a long history of human consumption—ATG is likely to have a favorable safety profile, which may facilitate its clinical development. Second, the multi-target nature of ATG, as revealed by our multi-omics analysis, aligns with the complex pathophysiology of RF, suggesting that it may offer advantages over single-target agents. Third, the observed non-significant trends toward improved efficacy when ATG was combined with IRB hint at the possibility of using ATG as an adjunctive therapy to enhance existing treatments. However, formal synergy studies with appropriate experimental designs and larger sample sizes are needed to confirm whether true synergistic interactions exist. Despite these promising attributes, several challenges must be addressed to advance ATG toward clinical application. First, as a lipophilic lignan, ATG may exhibit limited oral bioavailability due to poor aqueous solubility and extensive first-pass metabolism, which could restrict its systemic exposure and therapeutic efficacy. Second, the metabolic stability of ATG and its potential interactions with drug-metabolizing enzymes warrant systematic investigation, as these factors could influence both its pharmacokinetic profile and the risk of drug-herb interactions when co-administered with conventional medications such as IRB. Third, while *F. arctii* has a long history of human consumption, the safety profile of purified ATG at pharmacologically active doses requires rigorous evaluation in preclinical toxicity studies and eventual clinical trials. Future studies should therefore incorporate comprehensive pharmacokinetic assessments, formulation strategies to enhance bioavailability, and systematic evaluation of potential drug interactions to fully establish the translational feasibility of ATG for RF treatment.

**FIGURE 15:**
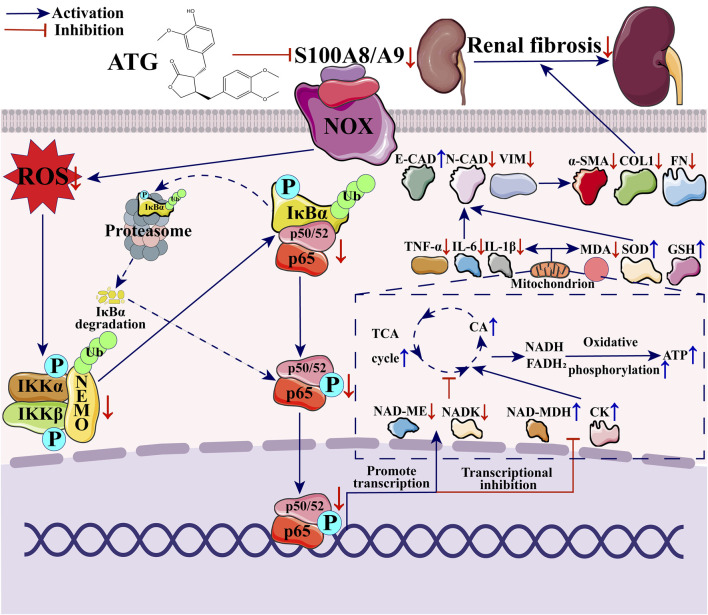
Schematic diagram illustrating the mechanism by which ATG ameliorates RF via regulation of the S100A8/A9/NOX/NF-κB signaling pathway, TCA cycle, and oxidative phosphorylation dysfunction, thereby suppressing inflammation and oxidative stress-driven EMT.

Our study has limitations. It remains a small-sample preclinical investigation; future work should advance to large-sample studies and clinical trials evaluating ATG for RF. Optimal dosing and administration strategies for ATG and IRB combination therapy require further investigation. Moreover, we acknowledge that the parameters used in this study to assess TCA cycle and oxidative phosphorylation function—including ATP levels, NAD^+^/NADH-related enzyme activities and CA content—are indirect indicators of mitochondrial metabolic activity. While these assays are widely employed and accepted as reliable surrogates for assessing metabolic pathway function, we recognize that more direct measurements would provide stronger evidence. Future studies should therefore incorporate complementary approaches such as assessment of mitochondrial membrane potential using fluorescent probes, real-time measurement of oxygen consumption rate using extracellular flux analysis, and specific activity assays for individual electron transport chain complexes. These direct functional assessments would further validate our current findings and provide deeper mechanistic insights into how ATG modulates mitochondrial bioenergetics to ameliorate RF. Additionally, we acknowledge that the detection of ROS using the DCFH-DA probe has inherent limitations. This probe is susceptible to auto-oxidation and non-specific oxidation, and it does not distinguish between different ROS species, such as superoxide anion and hydrogen peroxide. While DCFH-DA remains a widely used and accepted method for initial screening of oxidative stress, we recognize that more specific assays would provide stronger evidence. Future studies should therefore incorporate complementary approaches such as MitoSOX staining for mitochondrial superoxide detection and Amplex Red assays for quantitative measurement of hydrogen peroxide. These targeted assays would allow for more precise characterization of the specific ROS species involved and their subcellular origins, thereby further strengthening the mechanistic conclusions regarding ATG’s antioxidant effects. Furthermore, we acknowledge that the interpretation of EMT based on changes in classical markers such as E-cadherin, N-cadherin, and vimentin has inherent limitations. As increasingly recognized in the field, the expression of these markers can change independently of true lineage transition, and their modulation does not necessarily indicate a complete phenotypic conversion of epithelial cells into mesenchymal cells (Zhang C. X. et al., 2025; Zhu et al., 2025). While these markers remain widely used and accepted as indicators of epithelial plasticity and fibrogenic activity in RF research, we recognize that more definitive evidence would require lineage-tracing approaches or single-cell transcriptomic analyses to track the actual fate of tubular epithelial cells during fibrogenesis and following therapeutic intervention. Future studies should therefore consider employing genetic lineage-tracing models or high-resolution single-cell technologies to directly assess whether ATG truly inhibits EMT-driven cell fate transition or primarily modulates marker expression without altering cellular identity. Another limitation pertains to the relevance of the *in vitro* dosing regimen. The high-dose ATG concentration used in our cell experiments may exceed physiologically achievable plasma or tissue concentrations following *in vivo* administration. While this concentration was selected based on cell viability assays and preliminary experiments to ensure detectable biological effects, we acknowledge that it may not directly reflect the actual drug exposure levels in the kidney tissue of ATG-treated rats. Pharmacokinetic studies measuring plasma and tissue concentrations of ATG following oral or intravenous administration would be essential to establish the translational relevance of our *in vitro* findings. Future investigations should therefore incorporate detailed pharmacokinetic profiling to determine the clinically relevant concentration range and to validate whether the mechanisms identified *in vitro* operate at therapeutically achievable drug levels. Regarding the PAQ experiments, we acknowledge that the concentration used in our study is higher than reported IC_50_ values for S100A8/A9 inhibition. While this concentration was selected based on systematic cell viability assays and preliminary experiments to ensure detectable target inhibition, the relatively high concentration raises considerations regarding potential off-target effects. However, several observations support the functional relevance of PAQ-mediated S100A8/A9 inhibition in our experimental context. First, PAQ treatment alone recapitulated the key anti-fibrotic effects of ATG across multiple readouts, including suppression of fibrotic markers, inhibition of NF-κB phosphorylation, and attenuation of inflammatory and oxidative stress responses. Second, the lack of additive effects when ATG and PAQ were combined suggests that both agents act through overlapping mechanisms involving the S100A8/A9 axis. Third, the consistent downregulation of S100A8/A9 expression and its downstream pathways following PAQ treatment aligns with the expected pharmacodynamic effects of S100A9 inhibition. Nevertheless, we acknowledge that future studies incorporating detailed dose-response analyses and selectivity profiling across related protein families would further validate the specificity of PAQ at the concentrations used. In summary, our results indicate that ATG ameliorates EMT-driven RF by regulating energy metabolism disruptions via the S100A8 axis, thereby suppressing inflammation and oxidative stress (Figure 15).

## Conclusion

This study demonstrates that ATG exhibits significant nephroprotective effects and therapeutic efficacy in ameliorating RF. The underlying mechanism primarily involves the regulation of the S100A8/A9/NOX/NF-κB signaling axis, through which ATG modulates impairments in the TCA cycle and oxidative phosphorylation, thereby suppressing inflammation and oxidative stress-driven EMT and ultimately attenuating fibrotic progression. Our work provides a robust theoretical foundation for future interventions and prevention strategies targeting RF, supports further development and application of ATG as a therapeutic agent, and contributes to the pharmacological exploration of TCM by elucidating novel mechanistic pathways.

## Data Availability

The datasets presented in this study can be found in online repositories. The names of the repository/repositories and accession number(s) can be found in the article/supplementary material.

## Glossary

α-SMA: α-smooth muscle actin
AKI: acute kidney injury
ATG: arctigenin
ATP: adenosine triphosphate
BUN: blood urea nitrogen
CA: citric acid
CK: creatine kinase
CKD: chronic kidney disease
ECM: extracellular matrix
EMT: epithelial-mesenchymal transition
ESRD: end-stage renal disease
GO: gene ontology
GSH: glutathione
HE: hematoxylin-eosin
IκBα: inhibitor α of NF-κB
IKKβ: IκB kinase β
IL-6/1β: interleukin-6/1β
IRB: irbesartan
KEGG: Kyoto encyclopedia of genes and genomes
MDA: malondialdehyde
NAD-MDH: NAD-malate dehydrogenase
NAD-ME: NAD-malic enzyme
NADK: NAD kinase
NF-κB: nuclear factor-κB
NOX2/4: NADPH oxidase 2/4
PAQ: paquinimod
PBS: phosphate buffered saline
RF: renal fibrosis
ROS: reactive oxygen species
RT-qPCR: quantitative real-time reverse-transcription PCR
S100A8/A9: S100 calcium binding protein A8/A9
SCr: serum creatinine
SEM: standard error of the mean
SOD: superoxide dismutase
SUA: serum uric acid
TCM: traditional Chinese medicine
TGF-β1: transforming growth factor-β1
TNF-α: tumor necrosis factor-α
UUO: unilateral ureteral obstruction.
